# Rapid Determination of SiO_2_ Shell Thickness on Au Core Nanoparticles via Differential Centrifugal Sedimentation for SHINERS

**DOI:** 10.1021/acs.jpcc.6c01307

**Published:** 2026-06-08

**Authors:** Jacqui Everitt, Julia Fernández-Vidal, Khadija Younis, Shiying Qin, B. Layla Mehdi, Thomas O. Samuels, Alexander J. Cowan, Mathias Brust, Martin Volk, Laurence J. Hardwick

**Affiliations:** † Department of Chemistry, 4591 University of Liverpool, Crown Street, Liverpool L69 7ZD, U.K.; ‡ Stephenson Institute for Renewable Energy, University of Liverpool, Peach Street, Liverpool L69 7ZF, U.K.; § Department of Mechanical Materials and Aerospace Engineering, University of Liverpool, Brownlow Hill, Liverpool L69 3GH, U.K.; ∥ 263220 Johnson Matthey Technology Centre, Sonning Common, Reading RG4 9NH, U.K.

## Abstract

Consistent and reproducible synthesis of SiO_2_-coated Au nanoparticles, which are widely used as shell-isolated nanoparticles for enhanced Raman spectroscopy (SHINERS), is limited by the lack of rapid, routine methods to determine shell thickness and aggregation state. Here, differential centrifugal sedimentation (DCS) is demonstrated as a benchtop, collective-averaged technique capable of evaluating Au–SiO_2_ nanoparticles for SHINERS on time scales of under 10 min. Shell thicknesses determined by DCS show close agreement with transmission electron microscopy (TEM) images within the critical 3.5–5.5 nm range governing reliable pinhole free Raman enhancement, while simultaneously revealing early stage aggregation beyond the reach of UV–visible spectroscopy. By converting shell thickness and aggregation assessment into a rapid, decision-ready measurement, DCS enables routine quality control and synthesis-level optimization of reproducible particles for use in SHINERS.

## Graphical abstract

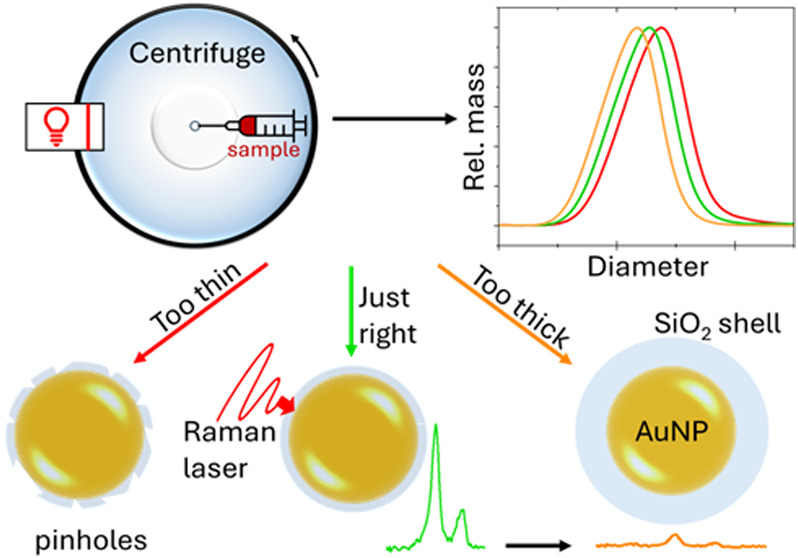

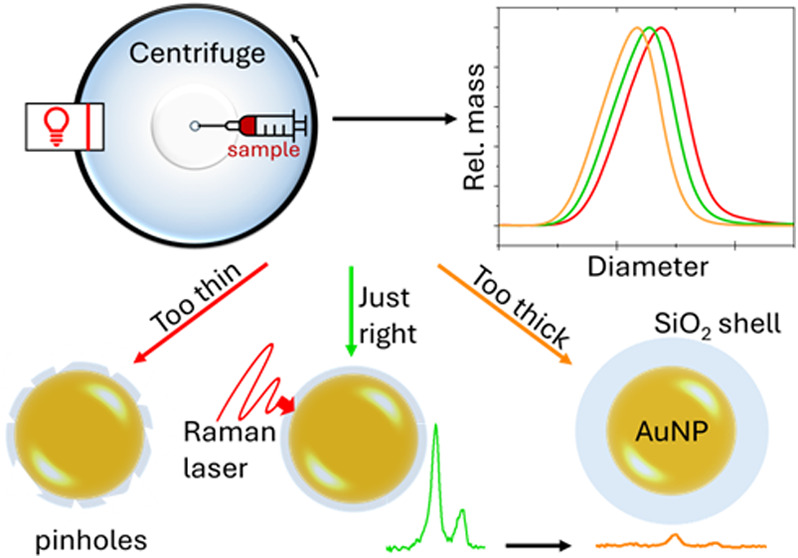

## Introduction

Shell isolated nanoparticle-enhanced Raman spectroscopy (SHINERS), first introduced by Li et al.^1^ in 2010, has catapulted Raman spectroscopy to the forefront of interfacial research as a facile method to probe the chemical composition of adsorbates and films with nanometre-scale resolution.^2,3^ Shell isolated nanoparticles (SHINs) allow for the localized surface plasmon resonance (LSPR) of the gold core to enhance the Raman signal without the gold being exposed to the analyte, system, or material under observation and, thus, can be applied to any substrate, unlike conventional surface enhanced Raman spectroscopy (SERS).^4–6^

Various types of systems, materials, and analytes have been investigated with SHINERS such as pesticide contamination on fruit,^1^ the detection of mannoproteins in yeast,^1^ determination of surface species on electrodes,^5,7–13^ as well as identifying species formed during surface corrosion,^14^ resolving reaction pathways within catalytic and electrocatalytic reactions.^5,15–21^ Immobilisation of SHINs on various substrates has enabled a range of *ex situ* and *in situ* studies within catalytic and electrochemical systems,^1,15,22,23^ highlighting the versatility of SHINERS as a spectroscopic investigative tool.^6,24^

Presently, the main challenge to overcome is the reliable and reproducible synthesis of SHINs that are pinhole free and enhance the Raman signal to an appreciable degree. In current literature, SHINs with a SiO_2_ shell thickness of 2–4 nm are generally used to achieve enhancement factors in the range of ca. 10^5^ to 10^8^.^12,14,16,22,25^ However, synthesis of 2–4 nm SiO_2_ shells that are pinhole free, can be challenging to achieve reproducibly, with even experienced laboratories having to periodically review the optimum synthesis conditions. The synthesis is highly reliant on the high-purity precursors being completely contaminant free and for the temperature and pH of the synthesis to be highly controlled and so large variations in shell thickness and pinhole occurrence can occur between each synthesis. Therefore, rapid and accessible quality control protocols are required to improve both success rates of SHIN synthesis and minimization of time for tuning the reaction conditions.

A prospective method to improve the analysis of synthesized SHINs is differential centrifugal sedimentation (DCS). DCS, first described in 1930,^26^ is a particle sizing technique that uses the sedimentation time of the particles in a fluid to determine the particle size and distribution. DCS has been shown to be an appropriate method for sizing nanoparticles and so using this method more information can be gained about the SHINs including average shell thickness,^27^ aggregation, and size dispersity.^29–31^ The DCS detector measures the extinction as particles pass through a laser beam as a function of time. The instrument then converts this information into a distribution in relation to the diameter of the nanoparticles, assuming spherical shape. Previously, DCS has been used to accurately determine the size distribution of spherical particles,^29–32^ as well as determining relative particle concentration in different samples.^32^ Furthermore, DCS has been used to follow protein corona formation on nanoparticles.^27,28,33,34^ When proteins adsorb to the nanoparticles, both their mass and their effective density change, which strongly affects the sedimentation time, and can even show the difference between physiosorbed and chemisorbed proteins. And, using similar protocols, DCS can be used to measure a polymeric shell,^34^ or inorganic coatings such as SiO_2_,^4^ on particles of a known density and diameter.

Herein, shell thicknesses determined by DCS are shown to be in close agreement with transmission electron microscopy (TEM) images within the critical range governing optimum and reliable pinhole free Raman enhancement, while concurrently revealing early stage aggregation, thus delivering a rapid quality control methodology, which is readily accessible.

## Experimental Section

### Materials

Gold­(III) chloride trihydrate ≥99.9% trace metals basis (HAuCl_4_·3H_2_O), (3-aminopropyl)­trimethoxysilane 97% (APTMS), sodium silicate solution extra pure (∼27 wt % SiO_2_), sulfuric acid 99.9% (H_2_SO_4_), sucrose (99.5%) were all used without further purification (Sigma-Aldrich). Trisodium citrate dihydrate (99%), 37% hydrochloric acid (HCl), 95% sulfuric acid (H_2_SO_4_), and 70% nitric acid (HNO_3_) were used without further purification (Fisher Scientific).

### Synthesis of Shell Isolated Nanoparticles

SHINs were synthesized in accordance with Li et al.^1,35^ who modified the method reported by Liz-Marzan et al.^36^ in order to limit shell thickness. The synthesis can be separated into two parts; gold nanoparticle synthesis and shell synthesis. Prior to all syntheses, all glassware and apparatus (e.g., magnetic stirrer bars, stoppers, water bottles, etc.) were cleaned using aqua regia (HCl/HNO_3_ 3:1), piranha solution (10 vol % H_2_SO_4_ in water), and boiled in ultrapure water (Milli-Q, 18.3 MΩ cm). See Supporting Information for further details on exact conditions and protocols. A simplified scheme can be found in the Supporting Information (Scheme S1).

### Synthesis of Gold Nanoparticles

200 mL of AuNP suspension were synthesized using the sodium citrate reduction method.^37,38^ Briefly, 2.43 mL of HAuCl_4_·3H_2_O 1 wt % was mixed under rapid stirring with 200 mL of ultrapure water (Milli-Q, 18.3 MΩ cm). The solution was then placed into a 100 °C water bath (i.e., under constant boiling) with rapid stirring. Once the solution was under reflux for at least 45 min, but no longer than 1 h, 1.44 mL of 1.14 wt % (38.8 mM) trisodium citrate dihydrate solution was rapidly added to obtain AuNPs of approximately 50 nm in diameter.

### Coating Gold Nanoparticles with a Silica Shell

An aliquot of the AuNP suspension (30 mL) was added to a round-bottom flask (100 mL) and was brought to room temperature. 410 μL of a 0.15 mM (3-aminopropyl)­trimethoxysilane (APTMS) solution was added slowly dropwise into the rapidly magnetic stirring AuNP suspension and was left to stir at room temperature for at least 30 min but no longer than 45 min. 3.6 mL of a premade 2 vol % of a sodium silicate solution at pH 10.2 (adjusted with 0.5 M H_2_SO_4_ immediately before addition to the vessel) was added to the AuNPs under rapid stirring. The resulting solution was left to stir at room temperature for 3 min before the vessel was moved to a 97 °C water bath with continued rapid stirring. One mL samples were taken at regular intervals and put into an ice bath. The samples were then put into a centrifuge (5500 rpm, 15 min) and the supernatant was removed. The particles were then resuspended using ultrapure water (Milli-Q, 18.3 MΩ cm) and centrifuged again, removing the supernatant when completed. Pinhole and enhancement tests as described in the Supporting Information were carried out for each sample to ascertain the suitability of the Au–SiO_2_ NPs for SHINERS following the protocol described by Li et al.^1^ where the SHINs are drop-cast and dried on Si and Au wafers (Figures S1 and S2).

### Raman Microscopy

Raman Microscopy (InVia, Renishaw) using a 632.8 nm laser (4 mW) with a single acquisition time of 10 s to collect spectra for pinhole and enhancement testing. To limit the buildup of particles (Figure S1) and increase the dispersity, a vacuum was applied to the SHINs that were drop-cast onto the Si and Au wafers for pinhole and enhancement testing, respectively (Figure S2). After determining which synthesized SHINs were pinhole free and enhancing via Raman spectroscopy testing using pyridine (see Figures S3 and S4),^1,7,9,39,40^ DCS, UV–vis, and TEM were performed to determine the shell thickness.

### Differential Centrifugal Sedimentation

The hollow disc of the centrifuge (CPS Instruments, Inc.), Figure S6, was first washed with ultrapure water (Milli-Q, 18.3 MΩ cm) followed by ethanol, a gradient of sucrose was made inside the disc while rotating at the maximum speed (24,000 rpm) using varying volumes of 8 and 24 wt % stock solutions of sucrose in ultrapure water (Milli-Q, 18.3 MΩ cm). 0.5 mL of dodecane was added to ensure evaporation of the water in the sucrose gradient does not occur. The gradient was left to settle in the DCS disc while at the maximum speed for at least 2 h. After the gradient settled, 0.05 mL of polyvinyl chloride (PVC) particles (10 mM PVC content, 0.263 μm diameter) calibration standard solution was injected into the DCS disc. Once calibration was complete, 0.05–0.1 mL of the AuNP or SHIN suspension (∼0.3 mM Au content) was injected into the DCS disc. Calibration is performed prior to each sample injection and each sample was measured three times, all while under the maximum rotation speed. During the DCS measurement the injected sample has to sediment through the sucrose gradient and pass through the light beam (Figure S6a), which allows the recording of the nanoparticle size distribution, here shown as the mass distribution of the sample, Figure S6b.

### UV–Vis Spectrophotometry

A UV–vis Spectrophotometer (Thermo Scientific Evolution 201) background was measured using ultrapure water (Milli-Q, 18.3 MΩ cm) between 300 and 800 nm before the extinction of 0.5 mL of each SHINs or AuNPs suspension (0.3 mM) was measured between 300 and 800 nm. Scans were completed at a scan rate of 1000 nm min^–1^ at a bandwidth of 2 nm with an integration time of 0.03 s.

### Transmission Electron Microscopy

Five μL of the SHINs or AuNPs suspension (0.3 mM) was drop cast onto transmission electron microscopy (TEM) grids (holey carbon film on 300 mesh Cu) and dried under a gentle vacuum. Eight −15 images of each shell reaction time interval sample and AuNP sample was taken using a JEOL JEM 2100+ operating at 200 keV.

## Results and Discussion

### DCS to Evaluate Early Aggregation of SHINs

To assess the ability of differential centrifugal sedimentation (DCS) to detect subtle changes in particle size and aggregation state, citrate-stabilized Au nanoparticles (AuNPs) and SiO_2_-coated SHINs that were synthesized from the same citrate stabilized Au-suspension, were analyzed and compared by UV–vis measurements. Figure 1a depicts four images of representative samples that were used in the DCS and UV–vis analysis in Figure 1b,c, respectively, to exemplify the different types of sols. There appears to be no visual difference between the first three samples (going left to right), with the fourth sample (far right) appearing to be a more purple color. The observed color change (red to purple) is a visual indication of an increase in particle size or particle aggregation.^41^ Aggregation occurs when colloidal particles lose the electrostatic forces that keep them apart, allowing them to come into contact and combine, forming larger clusters or aggregates.^42^ A small amount of aggregation may not be visible, but as more SHINs agglomerate, the effective particle size increases, which alters the way the particles interact with light, thus causing a visual change—in this case, a purple color. Qualitative observation of aggregation, such as SiO_2_ bridges, are observed via TEM images (Figure S15).

**Figure 1:**
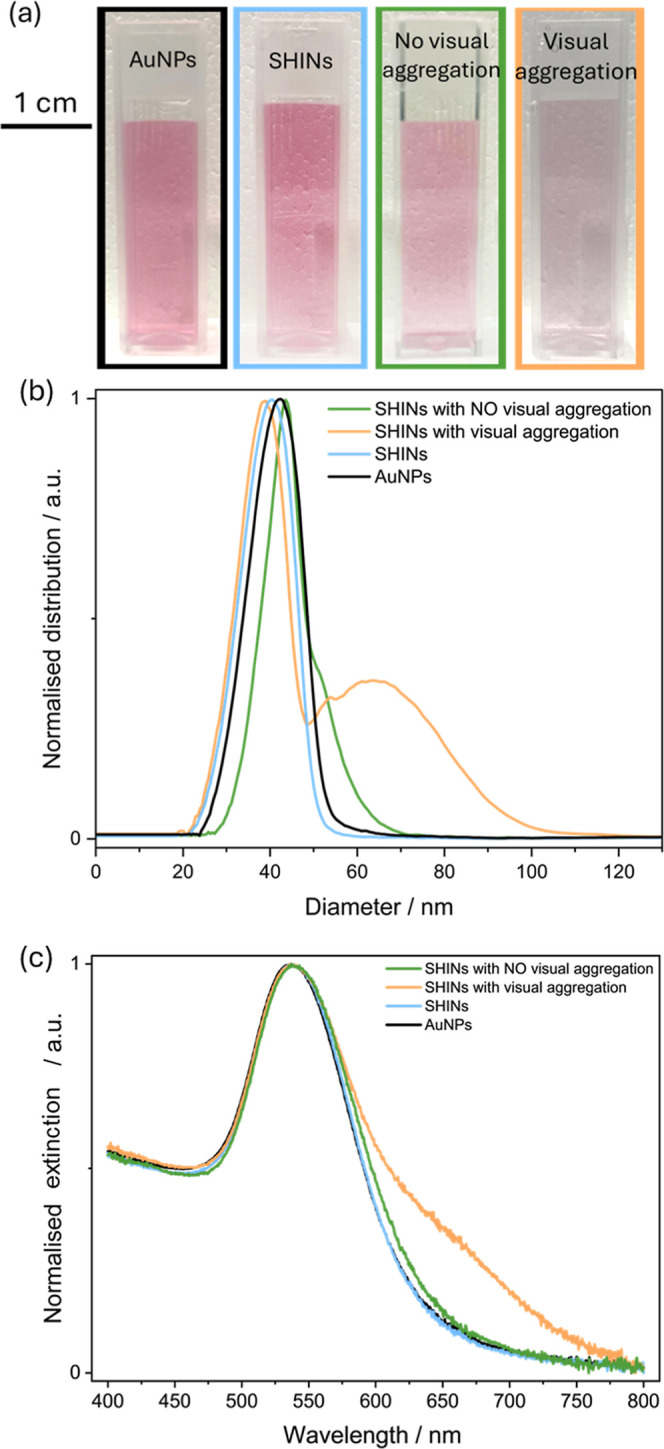
(a) Representative nanoparticle suspensions within cuvettes (b) DCS distributions, and (c) UV–vis spectra of samples of citrate-stabilized gold nanoparticles (black), SiO_2_ coated Au–SHINs (blue), and two aggregated SiO_2_ coated Au–SHINs samples synthesized from the same AuNP suspension (green and orange). The SHINs were synthesized under the same conditions.

**1:** Average Shell Thickness of SHINs Determined by DCS and TEM as a Function of Reaction Time for the Growth of SiO_2_ Shells on AuNPs^a^

	DCS	TEM
reaction time (minutes)	shell thickness (nm) (s.d. ±)	shell thickness (nm) (s.d. ±)
2	1.6 (0.1)	-
4	2.3 (0.1)	2.3 (0.1)
6	2.8 (0.1)	2.7 (0.2)
8	2.9 (0.1)	2.1 (0.4)
10	3.1 (0.1)	2.8 (0.4)
15	3.3 (0.1)	3.3 (0.5)
20	4.3 (0.1)	3.9 (0.4)
25	5.0 (0.1)	4.0 (0.5)
30	4.7 (0.4)	4.2 (0.4)
35	4.5 (0.1)	3.8 (0.2)
40	5.7 (0.2)	5.0 (0.3)
50	6.1 (0.7)	5.2 (0.4)
60	6.3 (0.4)	6.2 (0.4)
70	6.2 (0.1)	-
80	7.8 (0.1)	-
90	8.1 (0.8)	7.0 (0.9)
105	8.2 (0.5)	8.1 (0.8)
120	9.5 (0.8)	7.5 (0.8)
150	10.2 (0.1)	6.8 (0.7)
180	10.3 (0.1)	8.3 (0.5)
210	11.5 (0.7)	8.9 (0.6)
240	14.0 (1.3)	11.0 (2.1)

a The average shell thickness was determined by averaging the results of three separate SHINs syntheses.

In Figure 1b a narrow size distribution centered at 42.4 nm with a standard deviation of ±0.2 nm can be observed for the citrate-stabilized Au nanoparticles (black trace), indicating a low dispersity starting suspension (fwhm ∼ 14.3 nm). The peak shape remains similar for the SiO_2_ coated Au SHINs (blue trace), but with a slightly negatively shifted peak position to 40.6 (±0.1) nm, consistent with the reduced effective density of the core–shell particle, as discussed in detail below. The two other DCS distributions of SHIN samples (green and orange trace) show the appearance of a secondary peak at higher apparent diameters (51.7 (±0.4) nm and 63.5 (±0.6) nm, respectively). The emergence and broadening of these secondary features indicate the onset of aggregation. In particular, the fwhm of the peak at ∼63.5 nm was found to be ∼33 nm, which indicates that the aggregated particles have a greater polydispersity, compared to the SiO_2_ coated AuNP peak at 40.6 (±0.1) nm. The sample with the minor shoulder peak (51.7 nm) did not show any visual aggregation (Figure 1a third image) or clear variation in the UV–vis spectrum (Figure 1c). It shows that the sample of SHINs that had not aggregated (blue trace) and the aggregated sample (green trace) retain the red color that the uncoated AuNPs have, and so aggregation of this sample cannot be visually observed. UV–vis spectra can only show large to medium scale aggregation as secondary LSPR bands or loss of LSPR bands.^43^ In the UV–vis spectra in Figure 1c the visually aggregated SHIN sample (orange trace) shows a broad shoulder peak (643 nm) to the main LSPR peak (537.5 nm).^43^ This indicates a shift in the plasmonic response of the particles and increased particle size. However, for slight aggregation as shown in Figure 1c (green trace), UV–vis spectrophotometry does not show definitive aggregation and only shows a slight broadening of the LSPR peak.

DCS is therefore able to identify SHINs samples that have begun to aggregate, making them unsuitable for use in SHINERS measurements from both broadening of the plasmon band and inadequate dispersion at interfaces under investigation, see Figure S5a,b. Thereby DCS can be used as a facile quality control for the SHINs to identify samples in early stages of aggregation.

### Determination of SiO_2_ Shell Thickness on Au Core Using DCS

Increasing the thickness of the lower density SiO_2_ shell (2.65 g cm^–3^) onto the higher density Au core (19.3 g cm^–3^) decreases the effective density of the nanoparticles. Consequently, the DCS distribution curves appear to show an apparent decrease in nanoparticle diameter as shell reaction times increases from the point when the reaction vessel entered the water bath after adding silicate (Figure 2a,b**)**. By using the core–shell density model based upon the disparity in density between the Au core and SiO_2_ shell, as described in the SI, the real diameter and therefore the shell thickness can be determined, Figure 2c. The distribution curves in Figure 2a show no aggregation and the width of each curve is similar, as the fwhm values of all the distributions were 11.9 (±0.2) nm. The low error value indicates that the polydispersity across the samples is low, even with the increasing shell thickness. All synthesized SHINs within this study underwent characterization by DCS, and the results are summarized in Figure S9 and Table S1. The DCS traces are single peaks with low polydispersity, indicating the reproducibility of the synthesis method to produce aggregation free SHIN samples. The shell thickness that was determined by DCS has been represented as a function of the apparent diameter that was observed by the DCS (apparent diameter) as shown in Figure 2c. The line of best fit (red trace) can be used to predict the shell thickness of the SHINs when the DCS distribution has been obtained as it is essentially a calibration curve (in this case based on 48.6 nm diameter gold cores as determined by DCS).

**Figure 2:**
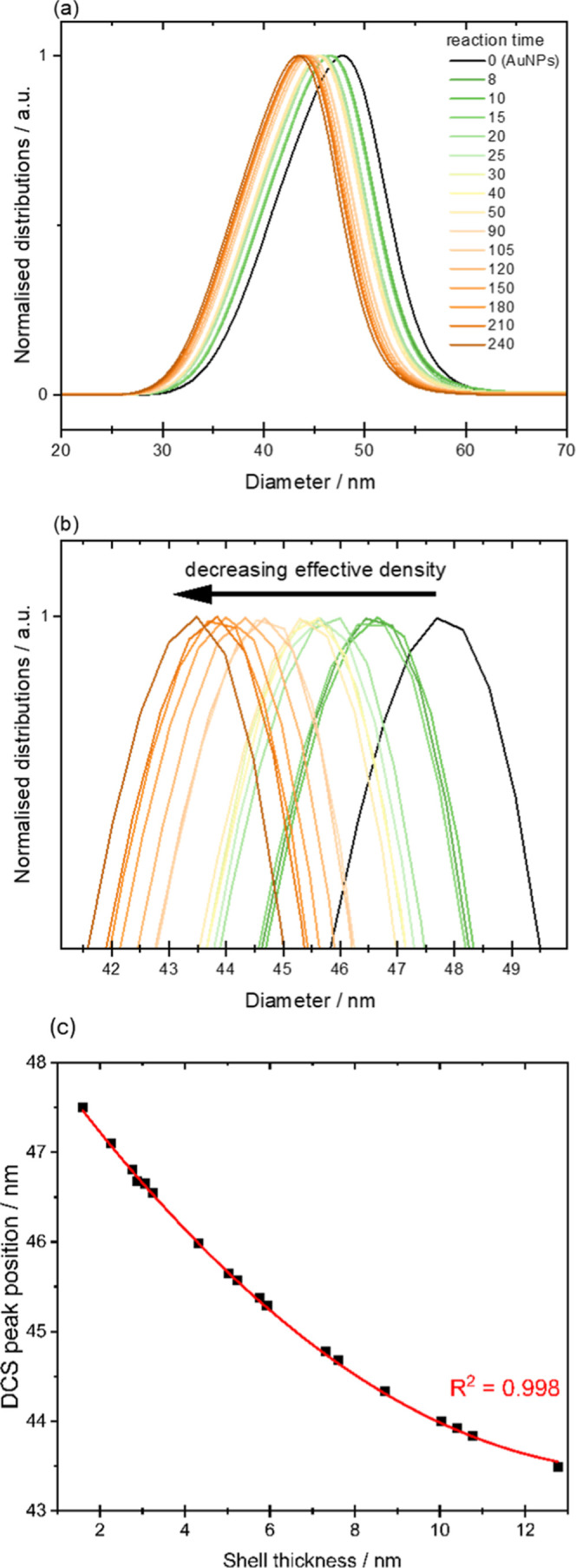
DCS results for a SHINs synthesis showing the full distribution curves (a) of the citrate stabilize AuNPs and Au–SiO_2_ SHIN samples for different time intervals, between 8 and 240 min of shell growth; and (b) zoomed in view of the peak maxima with an arrow showing the apparent decrease in diameter due to the decreasing effective density. (c) SiO_2_ shell thickness of synthesized SHINs where the Au core has a diameter of 48.6 nm compared to the apparent diameter of the SHINs as recorded by DCS (black points) with the curve of best fit (second order polynomial) and *R* ^2^ value shown in red. Equation S7 was used to determine the shell thickness at the DCS determined diameters shown in (c).

### Shell Thicknesses Obtained via DCS Compared to TEM

DCS is an accurate method to obtain average SiO_2_ shell thicknesses for the SHIN suspensions (see Introduction and Supporting Information Section 3); subsequently the DCS results are benchmarked against TEM in order to determine its general suitability as a reliable quality control method. Figure 3 shows TEM images across representative SiO_2_ shell reaction time intervals. Figure 3a shows a SHIN after 2 min of the synthesis where no distinguishable shell can be observed. After 15 min of synthesis a thin shell of (2.8 ± 0.6) nm thickness is observed in Figure 3b, shown through the lighter shade of the coating of the AuNP due to the less dense SiO_2_. The SiO_2_ coating gets visibly thicker with increasing time, as shown in Figure 3c–h, which increases from 30 min of shell reaction time to 240 min. At 30 min of shell synthesis the shell thickness, as calculated by TEM, was found to be 4.0 (±0.5) nm and at 240 min of shell synthesis was found to be 8.1 (±0.3) nm. Furthermore, as the shell thickness increases with reaction time, the overall uniformity of the SiO_2_ shell increases. This trend is observed in the TEM images from 30 to 240 min (Figure 3c–h), where the samples taken at 30, 50, and 90 min show more variable and uneven shells (Figure 3c–e), while longer reaction times yield thicker and smoother shells (Figure 3f–h).

**Figure 3:**
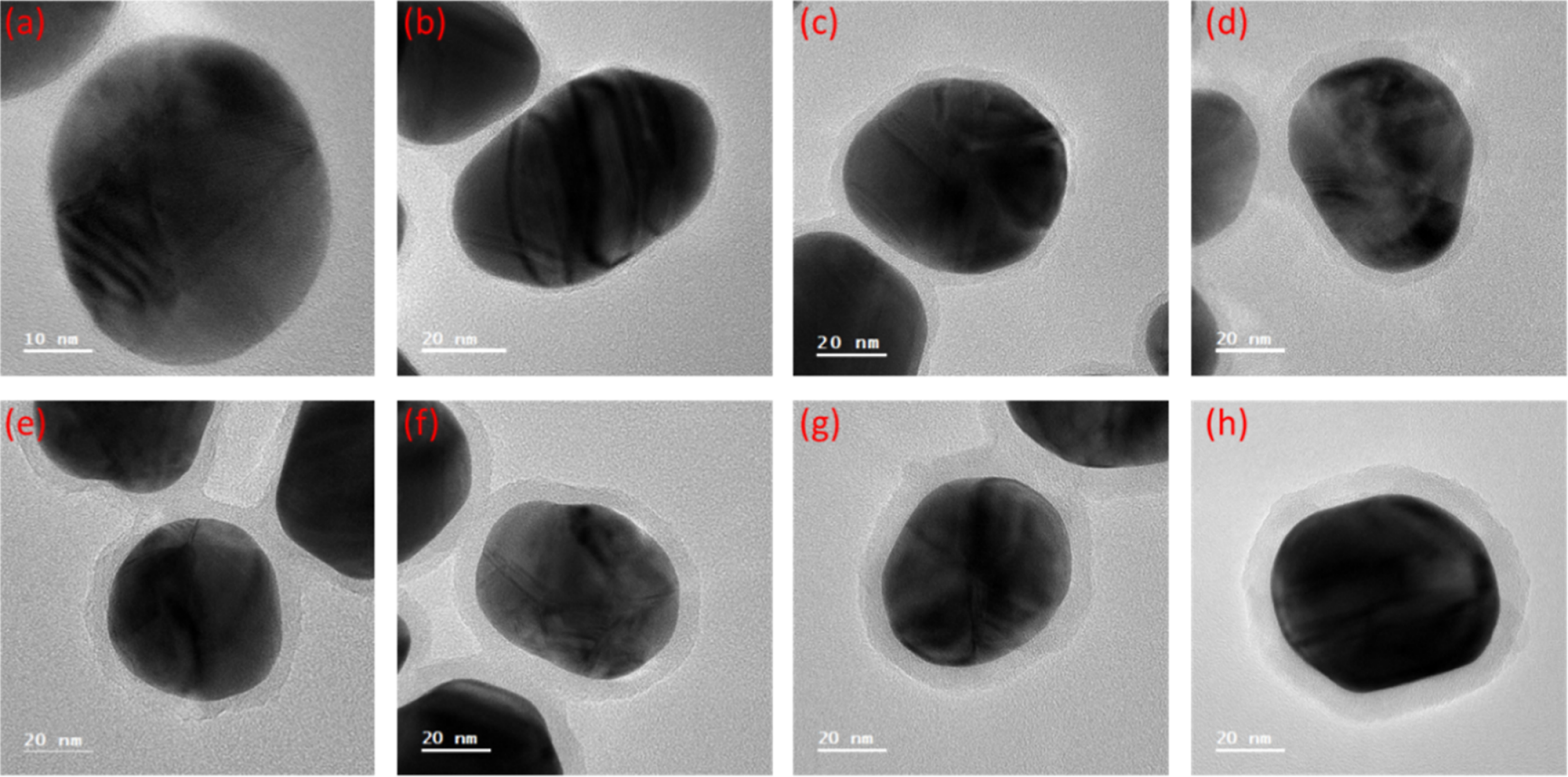
Selected TEM images of Au–SiO_2_ SHINs taken at different intervals during the synthesis of showing the increasing shell thickness: (a) 2, (b) 15, (c) 30, (d) 50, (e) 90, (f) 120, (g) 180, and (h) 240 min.

For example, at 50 min of shell growth the shell thickness for the SHIN shown in Figure 3d is 4.4 (±1.1) nm, whereas at 120 min the shell thickness for the SHIN shown in Figure 3f increases to 6.0 (±0.5) nm. The reduction in standard deviation with increasing reaction time reflects a more uniform shell thickness, consistent with the visually smoother shell observed at longer reaction times (this can also be observed in Figure S13). With the prolonged reaction time, the variation decreases due to the large overall buildup of the SiO_2_ shell, thus having a smoothing effect. The other shell syntheses and results of the shell thickness as determined by TEM can be found in Figure S14 and Table S2.

To get a better understanding of how the reaction time affects the shell growth and how the DCS obtained values compared to TEM, the values obtained for shell thicknesses from both methods for all the synthesized SHINs were averaged and are represented in Figure 4. In general, the results for shell thickness obtained using DCS are slightly higher than those obtained through TEM analysis. Over half of the error bars on the values obtained through TEM overlap with the corresponding DCS measurements, demonstrating strong agreement between the two methods and reinforcing confidence in the DCS-derived shell thickness, see Section 6 of Supporting Information and Table S4. After 105 min, shell thicknesses determined by DCS and TEM start to diverge. The shell thicknesses obtained by TEM for these higher reaction times do not follow the expected trend of increasing with time. For the discrepancy between DCS and TEM, particularly for the thicker shells, the results are consistent with increasing shell heterogeneity and a decrease in the effective shell density as the shell grows. Figure S13f, highlights TEM contrast variations that may indicate structurally different shell regions surrounding the Au core. Such heterogeneity would influence the sedimentation measured by DCS and therefore the apparent shell thickness obtained when a constant SiO_2_ density is assumed. As DCS measures the ensemble-average sedimentation behavior of the nanoparticle suspension, it is expected to be more sensitive to these effective density variations than local TEM measurements of individual particles.

**Figure 4:**
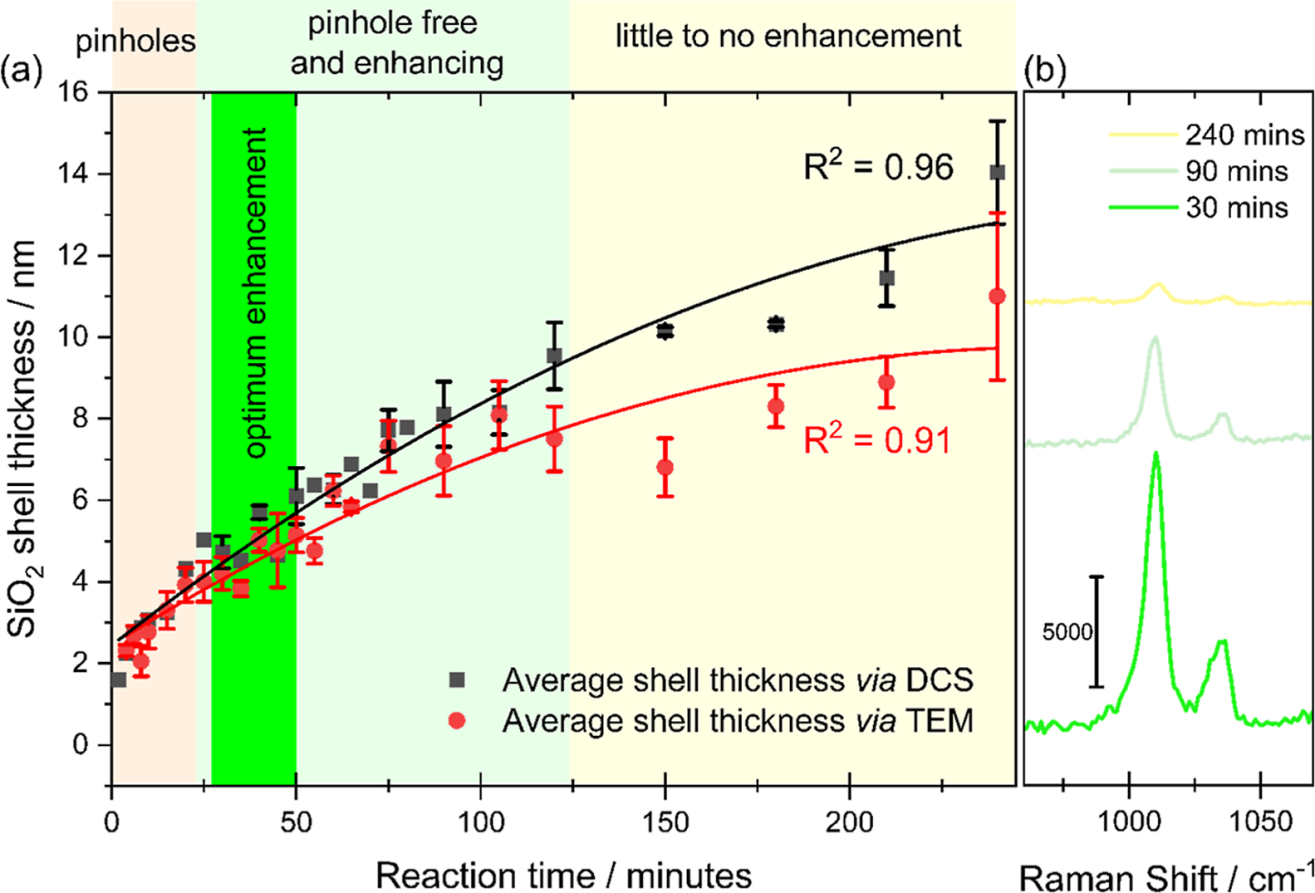
(a) Average shell thickness as a function of reaction time determined by DCS (black) and TEM (red), with lines of best fit to a polynomial of second order and corresponding *R* ^2^ values shown in the respective colors. The time intervals where SHINs have pinholes is orange, where the SHINs are pinhole free and enhancing is green (determined via the Raman enhancement of pyridine, shown in Figure S4), and where SHINs show little to no enhancement is yellow. The optimum range for SHINs with good enhancement is highlighted. (b) Raman spectra of pyridine band enhancement by SHINs from within the optimum reaction time (30 min, bright green trace), SHINs from outside the optimum reaction time with good enhancement (90 min, light green trace), and SHINs that show limited enhancement (240 min, yellow trace).

The SHIN sample across all the syntheses in this work with the greatest observed enhancement was the 30 min SHIN sample (see Figure S4), which had a shell thickness of 4.2 (±0.4) nm via TEM and 4.7 (±0.4) nm via DCS. However, as only one synthesis obtained pinhole free SHINs below 30 min, thinner shells that could potentially have greater enhancement could not be investigated. The average shell thickness for the 30 min samples is slightly above the typically reported values for the ideal range of shell thickness (2–4 nm) to achieve enhancement using a pinhole free shell, however, there are previous reports that used SHINs for SHINERS measurements that are thicker than 2 nm shells.^7,8,20,44,45^ The enhancement observed for the SHINs that have been shown to have a greater shell thickness than typically reported 2 nm could be due to the SHINs being well dispersed upon the planar gold substrate, generating more SERS active hot-spots per unit area.

The *R* ^2^ values for the polynomial fits of shell thickness vs reaction time are 0.96 for the DCS and 0.91 for the TEM analysis, highlighting the lower variability of the DCS analysis. The line of best fits are negative polynomials, which highlights that as the reaction time increases, the rate of shell growth decreases, due to the limiting factor of silicate solution concentration. The DCS line of best fit can therefore be used to tailor the shell thickness for future SHIN syntheses for the optimum enhancement, while avoiding pinholes. For example, a SHIN sample taken at 50 min, that was synthesized in accordance with the method described in the Supporting Information, could be predicted to have a shell thickness of 4.4–5.9 nm and so will yield SHINs that enhance the Raman signal for use in SHINERS.

The region of time that has been shown to reliably obtain pinhole free SHINs with the greatest enhancement in SHINERS measurements in this work is between 25 and 50 min, according to the Raman enhancement data of pyridine (Figure S4), and in this region all the data points for both DCS and TEM are in good agreement within the experimental accuracy and, thus, DCS is comparable to TEM. The optimum reaction time highlighted in green in Figure 4 indicates that the shell thicknesses of SHINs that can be routinely and reliably synthesized without pinholes that give sufficient enhancement in SHINERS measurements is between 3.5 and 5.5 nm.

This allows for a larger range of shell thicknesses to be used in SHINERS measurements which can be reliably and routinely made, as well as being more stable and easily used, whereas thinner 2 nm thick shells can be challenging to fabricate reliably and have a greater chance of aggregation occurring. Furthermore, it has been found that shells synthesized to up to 120 min which have a shell thickness of 7.5 (±0.8) nm via TEM and 9.5 (±0.8) nm via DCS can also show a degree of enhancement of the Raman signal of the typically investigated pyridine band.

UV–vis spectrophotometry has also previously been shown to be able to estimate particle diameters of citrate stabilized AuNPs by using the Haiss equation (see Supporting Information) by observing the LSPR. The LSPR occurs at a specific wavelength depending on size of the gold nanoparticle, as observed in the AuNP trace of Figure S16.^46,47^ When adding the SiO_2_ shell to the gold the dielectric constant in the immediate vicinity of the AuNP surface changes which causes a shift in the LSPR band observed by UV–vis for the SHIN samples.^48^ Although in principle it might be possible to use this shift for determining the shell thickness, quantification of the effect requires extensive calculations based on Mie theory and was not attempted here.^49^ In this case the LSPR will red shift to longer wavelengths as the shell grows, Figure S16b. What can be gained from the shift in the LSPR band is that as the SiO_2_ shell thickness increases the red shift is more pronounced and that UV–vis can give an indication on whether a coating has been made on the AuNPs. There is a correlation between increasing shell thickness and increasing red-shift (Figure S17), however, the extinction maxima for the UV–vis measurements of the 180 min and 240 min shell synthesis samples are very similar. The observed shell thickness obtained by DCS and TEM for the 240 min sample is ∼3 nm greater than the shell thickness for the 180 min sample (Table S3) so there is a limit to how reliable this correlation is, thus, UV–vis is of limited use for a precise analysis of the shell thickness of SHINs.

DCS has shown strong agreement with TEM for determining the shell thickness of SiO_2_–Au core SHINs. In particular, the SHIN samples exhibiting the greatest Raman enhancement of pyridine were the 30 min shell synthesis samples, for which DCS and TEM yield closely matching shell thicknesses. Furthermore, as the shell thickness increases with reaction time the agreement between DCS and TEM remains good and is essentially within the experimental uncertainty up to approximately 100 min, whereas beyond this time the results seem to increasingly diverge, with TEM reporting ∼20% smaller values. This divergence can be explained as DCS reflects the whole sample distribution, whereas TEM samples are a limited particle subset (15–20 particles).

In addition to the statistical advantage, DCS offers an additional practical benefit. Individual DCS measurements take less than 10 min per sample vs four hours for one sample via TEM (factoring in sample preparation and loading sample into the instrument). Additionally, to analyze the whole sample set by DCS is quick (ca. 30 samples per day, each measurement repeated three times) compared to TEM, for which it can take a couple of weeks to analyze data, thus increasing confidence in the shell thickness results obtained. Therefore, DCS can be used as a rapid alternative to TEM to determine the size of AuNPs and the shell thicknesses of SHINs. Given the critical role of the shell thickness of the SHINs, DCS provides a statistically robust assessment across large sample sets and an efficient alternative to TEM for routine characterization.

As previously discussed, UV–vis spectrophotometry can be used to identify large-scale aggregation through changes in the LSPR band but cannot determine whether early or subtle aggregation has occurred. DCS can show even small degrees of aggregation rapidly (under 3 min) by a definitive shoulder peak rather than the ambiguous broadening of the SPR band. As such, DCS can be used as a rapid method for assessing aggregation as well as SiO_2_ shell thickness determination.

Furthermore, DCS is a benchtop technique that does not require highly specialized training and is easily accessible. It offers a high resolution (*ca*. 0.1 nm) over a wide practical range from 2 nm to 80 μm, making DCS a useful tool for determining the shell thickness and the state of aggregation of SHINs, as compared to the time-consuming nature of TEM, which requires experienced practitioners and the limited sensitivity of UV–vis spectrophotometry.

## Conclusions

Differential centrifugal sedimentation (DCS) has been shown to be both a rapid and accurate benchtop method for determining the aggregation state and the average shell thickness of the gold core SiO_2_ particles widely used in the surface enhanced Raman spectroscopy method, SHINERS. DCS was compared to the more established methods of UV–vis spectrophotometry and TEM and was able to provide bulk sample analysis (10–100 s of μL) over a coating range of 2–14 nm. DCS shows greater accuracy than UV–vis, as well as overcoming the limitation of TEM of obtaining just a small set of images, typically <20 particles. DCS was used to tailor synthesis conditions to optimize the shell reaction time (and thus coating thickness) to deliver reliable SiO_2_–Au particles with a shell thickness between 3.5 and 5.5 nm that provide good and reliable Raman enhancement, without the presence of pinholes. More broadly, DCS supports the more routine synthesis of SiO_2_–Au particles for SHINERS and enables determination of early aggregation. This highlights the potential of DCS, not only for SHINs, but also for the analysis of nanoparticles with other coatings on a range of metal cores of varying diameter, which would make the method highly useful for the broader analytical research community.

## Supplementary Material



## Data Availability

800 TEM images of the samples were collected and analyzed. The TEM images are available at a repository, as well as the DCS measurements and excel spreadsheet used to determine shell thickness and can be found here: University Data Catalogue, Elements, TEM images of SHINs ID: 3042: 10.17638/datacat.liverpool.ac.uk/3042.
