# Supplementary material for: Rapid Determination of SiO2 Shell Thickness on Au Core Nanoparticles via Differential Centrifugal Sedimentation for SHINERS
Source: J Phys Chem C Nanomater Interfaces. 2026 Jun 8;130(24):8363–71. doi: 10.1021/acs.jpcc.6c01307 (PMC13288673; doi:10.1021/acs.jpcc.6c01307)
Supplement: Supplementary file 1 [file jp6c01307_si_001.pdf]

**Supporting Information**

# Rapid Determination of $SiO_2$ Shell Thickness on Au Core Nanoparticles *via* Differential Centrifugal Sedimentation for SHINERS

Jacqui Everitt[a,b], Julia Fernández-Vidal[a,b], Khadija Younis[a,b], Shiying Qin[c], B. Layla Mehdi[c], Thomas, O. Samuels[d], Alexander J. Cowan[a,b], Mathias Brust[a], Martin Volk[a], Laurence J. Hardwick[a,b *]

a Department of Chemistry, University of Liverpool, Crown Street, Liverpool, L69 7ZD, UK

b Stephenson Institute for Renewable Energy, University of Liverpool, Peach Street, Liverpool L69 7ZF, UK

c Department of Mechanical Materials and Aerospace Engineering, University of Liverpool, Brownlow Hill, Liverpool L69 3GH, UK

d Johnson Matthey Technology Centre, Sonning Common, Reading, RG4 9NH, UK

*Corresponding author: hardwick@liverpool.ac.uk

# 1. AuNP and shell synthesis

## 1.1. Glassware cleaning protocol

All glassware used was cleaned extensively before use whether for synthesis or electrochemistry. First, the glassware was filled with aqua regia (3:1 vol. $HCl:HNO_3$), swirled and emptied up to three times and then rinsed with high purity water (MilliQ, 18.3 MΩ) before being half filled with high purity water and vigorously shaken; repeated up to 10 times. This ensures that no metallic contaminants remain in the glassware. The glassware was then filled with 10 vol% piranha solution (5:1 $H_2SO_4:H_2O_2$ then made to 10 vol% with high purity water) and left overnight. The piranha solution was then emptied out, and the glassware was rinsed with high purity water before being half filled with high purity water and vigorously shaken; repeated up to 10 times. This removes all organic contaminants from the glassware. Finally, the glassware was filled with high purity water and boiled three times. After each boil the glassware was rinsed with high purity water before being half filled with high purity water and vigorously shaken up to 10 times. The boiling of the glassware serves two purposes: 1. To ensure that there is no aqua regia, piranha solution, or other [soluble] contaminants are present and 2. To functionalise the respective surfaces of the glass ware and stirrer bar such that adhesion of the Au nanoparticles is unfavourable during nucleation and growth of the particles and shell.[1] Furthermore, all glassware was replaced every 2 – 3 months due to the build-up of silica on the interior surface which can lead to irreproducible results.

## 1.2. SHIN Synthesis Protocol

SHINs were synthesised in accordance with Li *et al*.[2, 3] which is a modified version of the method reported by Liz-Marzan *et al.*[4] The modifications made by Li *et al.* were to increase the temperature of reaction from room temperature to 95 °C and to limit the concentration of $SiO_2$ to limit shell thickness. Prior to all syntheses, all glassware and apparatus (e.g., magnetic stirrer bars, stoppers, water bottles, etc) were cleaned as described in **1.1**. The room temperature was maintained between 26 and 29 $^{o}$C

with a humidity of ~20%. SHIN synthesis can be separated into two parts: gold nanoparticle (AuNP) synthesis and the coating of the AuNPs with a silica shell.

### 1.2.1. Gold Nanoparticle Synthesis

AuNPs were synthesised using the sodium citrate reduction method.[5, 6] A stock solution of $HAuCl_4.3H_2O$ (1 wt.%) was made in a volumetric flask (100 mL) using high purity water (MilliQ, 18.3 MΩ) this solution was made at least 3 days before use to ensure complete mixing and hydration of the $HAuCl_4$. 2.43 mL of the $HAuCl_4$ stock solution was mixed under rapid stirring with high purity water (200 mL) in a two-neck round bottom flask (250 mL) to get a final solution of 0.30 mM $HAuCl_4.3H_2O$. This solution was left under rapid magnetic stirring for at least 45 minutes, but no longer than 1 hour, to ensure thorough mixing and complete hydration of the $HAuCl_4$. The solution was then placed into a 100 °C water bath (i.e. under constant boiling) with rapid stirring. Once the solution was under reflux (ca. 45 minutes), trisodium citrate dihydrate (1.44 mL, 1.14 wt%, 38.8 mM) was rapidly added, the solution went from a straw yellow colour to colourless to black to purple then finally to a deep red solution over the course of ~5 minutes after the addition of the citrate. The solution was left under reflux and rapid magnetic stirring for at least 45 minutes, but no longer than 1 hour, after the deep red colour of the solution had been reached. The reaction vessel was removed from the water bath, and the deep red solution was left to stir overnight at room temperature. The solution was then stored in a refrigerator at ~5 °C.

### 1.3.2. Coating Gold Nanoparticles with a Silica Shell

An aliquot of the AuNP solution (30 mL) was added to a round bottom flask (100 mL) and was brought to room temperature. A simplified schematic of this process can be seen in **Scheme S1**. APTMS (410 µL, 0.15 mM) was added slowly drop wise into the rapidly magnetic stirring AuNP solution and was left to stir at room temperature for at least 30 minutes, but no longer than 45 minutes. The APTMS replaces the citrate stabilisers on the AuNPs. If the citrate is not fully displaced, then pinholes will occur and so stirring for 30 minutes helps to ensure the full displacement. An aqueous solution of sodium silicate was prepared by adding 2 mL of extra pure ~27 wt% sodium silicate solution to ~ 70 mL of high purity

water (MilliQ, 18.3 MΩ) in a 100 mL volumetric flask. The pH of this solution was adjusted to ~ pH 10.2 using $H_2SO_4$ (0.5 M) by adding the $H_2SO_4$ dropwise under vigorous stirring. The pH adjusted $NaSiO_2$ solution (3.6 mL, 2 vol%) was added to the AuNPs under rapid stirring and left to stir at room temperature for 3 minutes before being moved to a 97 °C water bath under constant stirring. 1 mL samples were taken at regular intervals and put into an ice bath. The samples were then put into a centrifuge (5500 rpm, 15 minutes) and the supernatant was removed. The particles were then resuspended using high purity water and centrifuged again, removing the supernatant when completed.

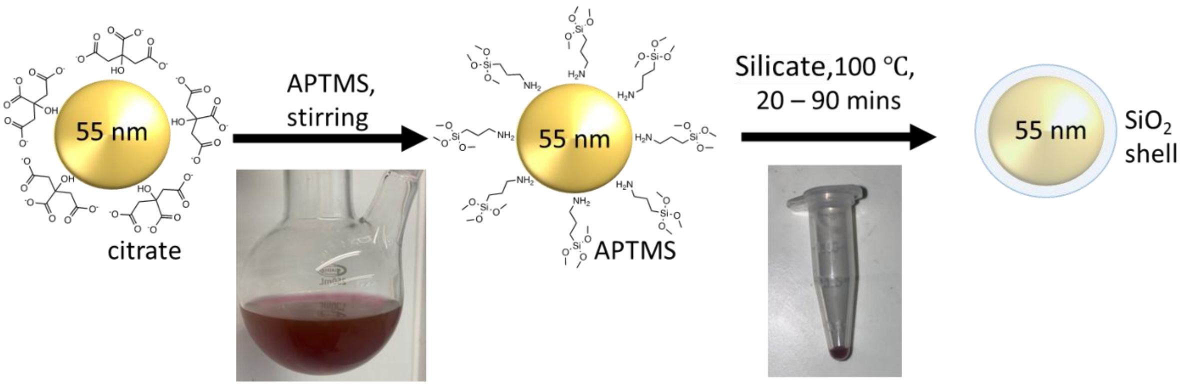


**Scheme S1.** APTMS is added to an aliquot of the red AuNP suspension where the displacement of the citrate stabilisers occurs. This occurs due to the more favourable bonding of the N tail of the APTMS than the OH of the citrate which derives from amines having a larger complexation constant to gold than the citrate hydroxy groups.[4] Once the displacement with the APTMS is completed, the silicon dioxide can form bonds with Si-O of the APTMS and then polymerise to form the shell.[7]

### 2.1. SHIN Pinhole Testing Procedure

A sample of the SHINs was diluted in 0.5 – 1 mL of high purity water (MilliQ, 18.3 MΩ) to create a dispersion of the SHINs in solution. 20 µL of this dispersion was drop cast on a silicon wafer. The wafer was put into a desiccator with no desiccant under a gentle vacuum until dry (*ca*. 30 minutes), after which they were removed. The vacuum is used to reduce the coffee ring effect that occurs when the colloidal liquid dries, see **Figure S1**. The phenomenon arises in cases where the contact line of the drop

is pinned to its starting position, as is the case for water drops on most surfaces. Evaporating water molecules from the edges of the drop are replenished by water from within the drop, creating a flow of suspended particles towards the edges of the drop, **Figure S1**.[8]

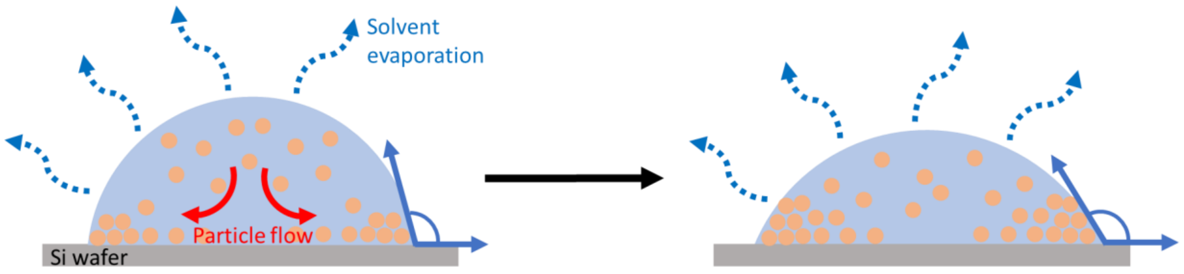


**Figure S1.** Coffee ring formation mechanism of colloidal gold droplet on a silicon wafer showing the colloidal nanoparticle flow and the solid-liquid contact angle as the solvent, water, evaporates.

Under normal drying conditions this leads to a large excess of suspended particles at the edges (**Figure S2a**), which manifests as a thick ring upon drying.[9] As the solvent, in this case water, evaporates the solid-liquid contact angle decreases, indicating a decrease in volume in the droplet. When this contact angle reaches a critical point the deposition of the particles starts to occur independent of the particle flow and therefore the centre of the coffee ring can still contain particles. Using a gentle vacuum allows for a more uniform and quicker drying process which limits the build-up of particles at the edge (**Figure S2b**). The vacuum increases the speed of solvent evaporation which increases the concentration of colloidal particles in the droplet. This decreases the rate of particle flow and, thus, causes a more uniform deposition of the particles, decreasing the coffee ring effect but not fully preventing it.

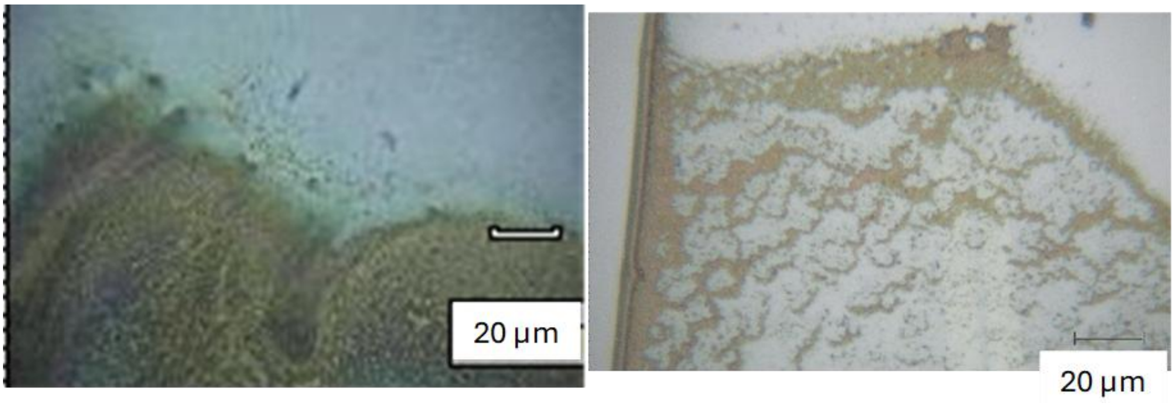


**Figure S2**. Coffee ring effect of 20 μL of colloidal AuNPs dried on a Si wafer when dried under atmospheric pressure (a) and under a vacuum (b).

To test for pinholes, 5 – 10 μL of 10 mM pyridine (in water) was dropped onto each wafer and a cover glass was placed on to ensure good contact and coverage of the wafer with the pyridine. The probe molecule, pyridine, was selected due to the distinct ring breathing and asymmetric stretching modes that occur at 1009 and 1035 $cm^{-1}$ respectively.[3, 10-12] Also, pyridine has a strong adsorption to gold but weak adsorption to silicon,[10] therefore, if the shell is pinhole free then no pyridine bands would be seen. Generally, the wafers were placed in a Renishaw inVia Raman confocal microscope, and several sites were tested a 633 nm wavelength laser, 4 mW laser power, 10 s acquisition with 1 accumulation.

Results of three different syntheses of SHINs are reported, and the pinhole test was conducted on each sample until pinholes were not found. To ensure that a representative sample of the SHINs were tested for pinholes, spectra of *ca.* 15 different spots across the sample were taken. If no pyridine bands were observed on the spectra, then the sample was said to be pinhole free. **Figure S3** shows an example of the pinhole test being performed on a sample of SHINs where the characteristic ring breathing and stretching modes of pyridine adsorbed onto gold can be observed at 1010 and 1035 $cm^{-1}$, respectively. When the synthesis time reaches 30 minutes, the pyridine bands can no longer be observed.

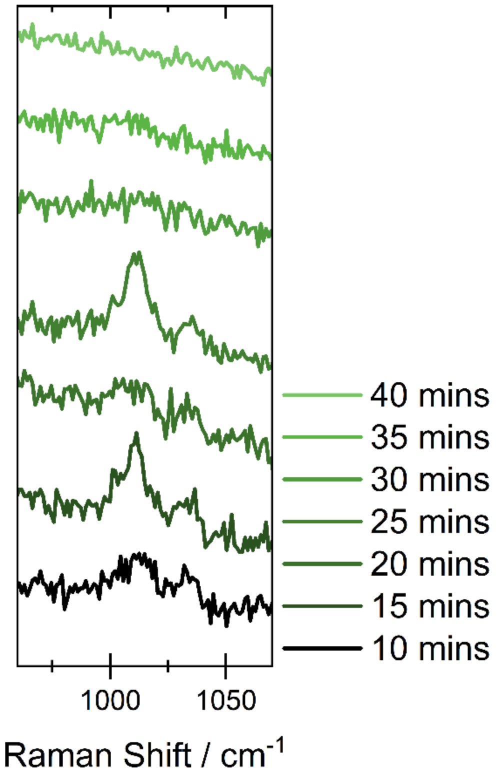


**Figure S3**. Pinhole test Raman spectra of SHIN samples taken out of the reaction mixture at various times during the synthesis drop cast on silicon wafers. Characteristic pyridine Raman bands can be seen at 1010 $cm^{-1}$ and 1035 $cm^{-1}$ up to 25 minutes of synthesis. These SHIN samples were taken from the third SHIN synthesis to be used as an example of the pinhole test.

### 2.2. SHIN Enhancement Testing Procedure

The method followed for this is the same as the pinhole test method except gold wafers are used rather than silicon wafers. The coffee ring effect is even more important when considering enhancement. Where the concentration of the SHINs is the greatest the enhancement of the Raman signal will be less due to the loss of the nanostructured properties. An ideal site for the greatest enhancement, colloquially known as a hot spot, is an area where the nanoparticles are dispersed enough that they retain the nanostructured properties but close enough to have overlapping SPRs.

The SHIN samples that were shown to have no pinholes then underwent Raman enhancement testing, **Figure S4**. To validate the Raman signal enhancing ability of the synthesised SHINs, the samples are drop cast onto gold wafers. In the first of three different syntheses, pinholes were found until 10

minutes of synthesis, thus **Figure S4a** shows enhancement testing of sample from 15 minutes onwards as these did not have pinholes. In the other two syntheses, pinholes were observed up until 25 minutes of shell synthesis and so the enhancement testing in **Figures S4b** and **S4c** show enhancement testing of samples from 30 minutes onwards as they do not have pinholes. In **Figure S4** all three syntheses show that the sample with the greatest observed enhancement is the 30 minutes shell growth sample. In **Figure S4a**, the samples at 15, 20, and 25 minutes should have thinner shells than the sample at 30 minutes and should therefore show a greater enhancement, but the intensity of the pyridine bands is much lower than at of the sample taken at 30 minutes. One explanation for the variation of the intensity of the pyridine peaks shown at 1010 and 1035 cm$^{-1}$ is due to the area of the sample taken; in other words, the variation could be due to a good SERS hotspot not being found for those samples and thus the best enhancement could not be observed. Another explanation could be that the particles in this sample could not have dispersed in the optimum way due to concentration, drying time, or agglomeration of particles. For all syntheses, the thicker the shell the weaker the enhancement is, as shown through the lower intensity of the pyridine bands.

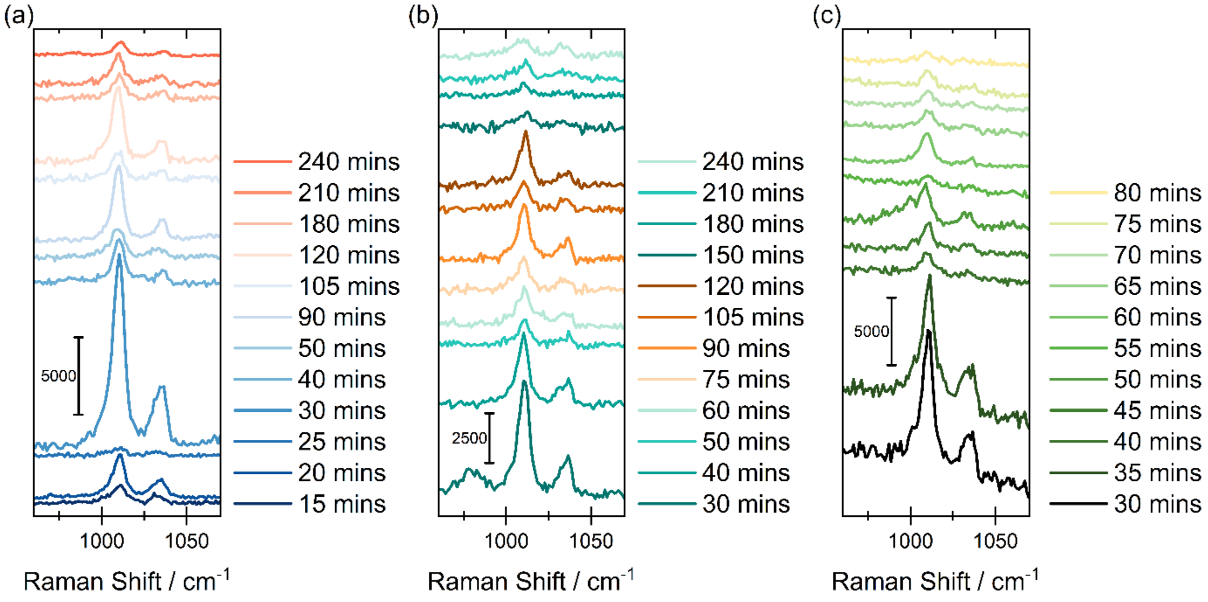


**Figure S4**. Enhancement test spectra of pyridine on gold wafers enhanced by drop cast SHINs from three different syntheses.

## 2.3. Effect of aggregation on enhancement

A sample of visually aggregated SHINs (as seen in **Figure 1a**) was deposited onto a gold wafer and images were taken of the resulting deposition (**Figure S5a**). The aggregated SHINs alter interparticle plasmon coupling and do not generate any enhancement of the Raman signals as observed in **Figure S5b** due to the absence of the characteristic Raman bands of pyridine at 1010 and 1035 $cm^{-1}$.

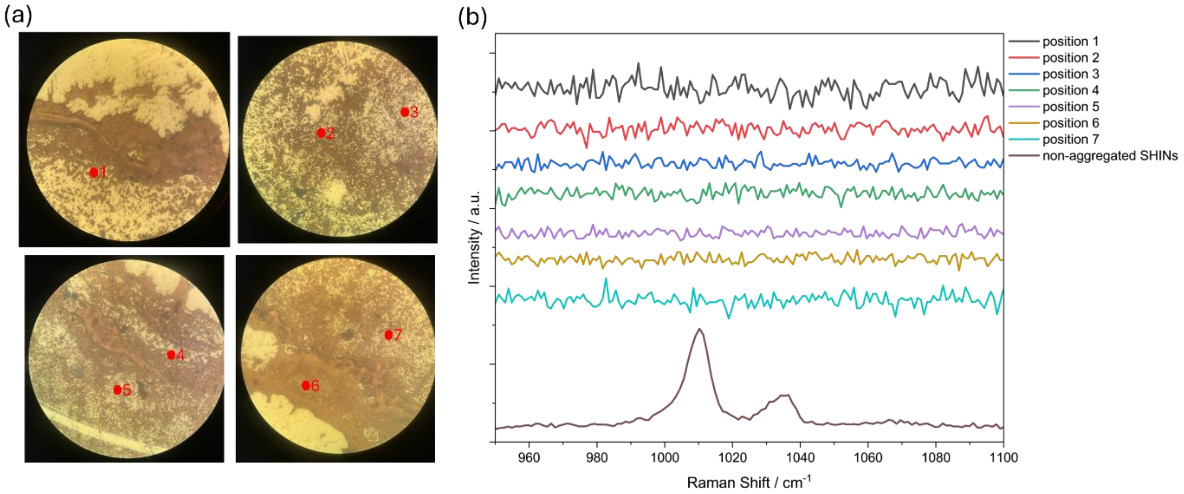


**Figure S5.** (a) Four optical images at x50 magnification of different areas of an aggregated SHIN sample deposited and dried on to a gold wafer with numbered red dots showing where enhancement testing was performed. (b) Raman spectra from seven different areas (indicated in (a)) across the aggregated SHIN sample dried onto a gold wafer, testing for enhancement with pyridine as a probe molecule, and the enhancement test of non-aggregated SHINs showing the two characteristic pyridine bands.

### 3.1. DCS apparatus, calculations, and theory expanded

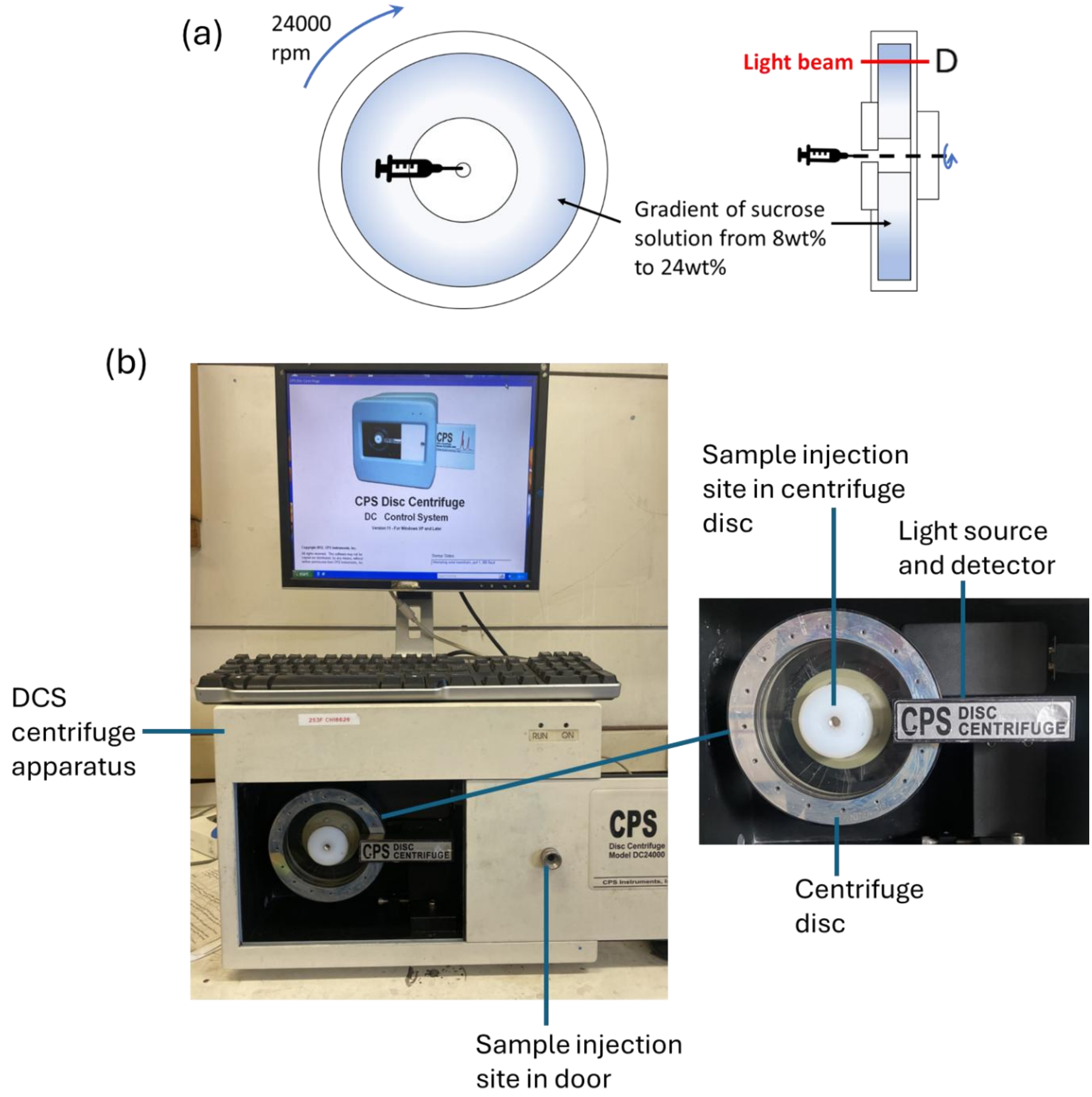


**Figure S6**. Schematic of differential centrifugal sedimentation apparatus (a) The front and side view of the hollow centrifuge disc with a gradient of sucrose inside. The light beam goes through the gradient to a detector, and its intensity is recorded by a detector (D) on the other side, reducing when nanoparticles pass through the beam. The injection site to the centrifuge is located at the centre axis to ensure the sample is injected at the same position each time. Disc centrifuge (CPS inc. disc centrifuge model DC24000) used for differential centrifugal sedimentation method (b) and close view of central disc (to the left).

**Theory behind DCS**

DCS can determine the size of nanoparticles by employing Stokes' law by measuring the time that particles take to sediment in a fluid of known viscosity and density over a known distance. Stokes' law describes the frictional force of nanoparticles within a fluid of known viscosity and density, however, it is limited to spherical-like particles with a very small Reynolds number, which is a dimensionless quantity that helps predict flow characteristics of a fluid. The particles must have a low Reynolds number so they can be assumed to behave like a liquid under laminar flow. Other assumptions needed to allow Stokes' law to describe the particle size using DCS is that the particles in the fluid do not interfere with each other, are uniform in composition, and move under laminar flow,[13] i.e. the particles flow in regular paths without fluctuations or mixing. As the technique must assume that all particles are completely spherical and have no interaction with each other there may be a small error or deviation in the results if the particles are not completely spherical. Deviations from spherical shape led to minor underestimations of the particle sizes. However, even for nanorods with an aspect ratio of 2 (length/width), the effective size is underestimated by only 5%, as per the DCS manufacturers guidance. The SHINs investigated here have even less deviation from spherical shape, see **Figures 3** and **S10 – S14**, and so the deviation will be even less than 5% and so is within experimental error. **Equation S1** shows the viscous force on a small sphere moving through a viscous liquid where $F_d$ denotes the frictional force acting on the interface between the fluid and the particle (N), μ is the dynamic viscosity (Pa.s), D is the diameter of the spherical object (m), and V is the flow velocity relative to the object (m $s^{-1}$

$$F_d = 3\pi\mu DV \qquad \textbf{(S1)}$$

When utilising DCS a centrifugal force is applied by rotating the DCS disc around the centre axis. The applied centrifugal force makes the sedimentation of even the smallest of particles occur a lot quicker and so accurate size distribution measurements can be taken. When a centrifugal force is applied, Stokes' law can be used to calculate the velocity at which the particle moves, **Equation S2**, where V is

the flow velocity (m $s^{-1}$), D represents particle diameter (cm), $\rho_p$ is the particle density (g $mL^{-1}$), $\rho_f$ denotes the fluid density (g $mL^{-1}$), G is the rotational acceleration (cm $s^{-2}$), and η is the fluid viscosity. For a centrifuge running at a constant speed and at a constant temperature, all parameters in **Equation S1** become constant and by using a traceable standard before every measurement, such as poly vinyl chloride (PVC) synthesised to a specific size (here: 0.263 µm),[14] then the time that the particles require for sedimentation to the detector can be converted to determine their size.

$$V = \frac{D^2 (\rho_p - \rho_f) G}{18\eta} \quad \textbf{(S2)}$$

In DCS, differential sedimentation is used, which means that only a small part of the distribution of particles is detected and measured at any one time during the sedimentation of the particles, **Figure S7a**. The detector initially reads zero extinction and, when particles enter the detector beam, in this case a monochromatic light, the extinction is increased and once all particles have passed through the beam the reading goes back to zero, **Figure S7b**.[15] Furthermore, larger particles sediment faster and reach the detector first, **Figure S7a**. Mie theory describes how a spherical particle interacts with electromagnetic waves (i.e. light) and is applied in DCS as different size particles will interact differently with the wave. Thus, Mie theory can be applied to the extinction data to convert them to the mass distribution with respect to particle size.[16] (**Figure S6b**).

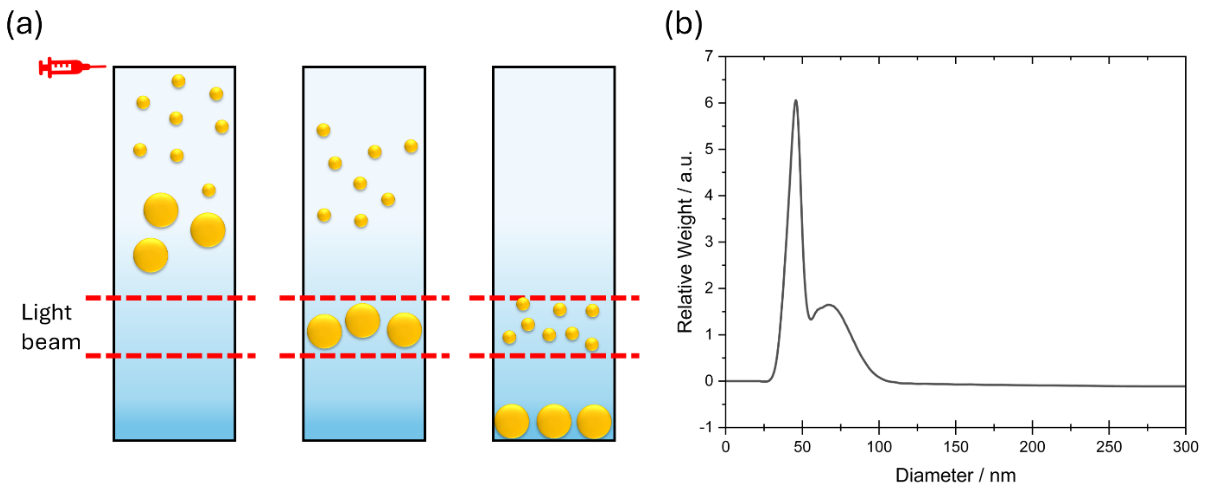


**Figure S7.** (a) Nanoparticles of varied sizes passing through the light beam with the larger particles passing through first causing an increase in light extinction from zero as shown in the distribution (b). The time-dependence of the absorbance can be directly converted into the size distribution of the particles, using **Equation S2** and Mie-theory, here shown as the mass-distribution. The secondary peak indicates that a mix of different sized particles are present.

However, if the particles are denser than the liquid, bulk settling of the particles can occur which means all information of the particle size distribution can be lost. To combat this the fluid used is often made up of a density gradient, for example, 8 wt.% aqueous sucrose to 24 wt.% aqueous sucrose. This stops bulk settling from occurring because there is a continuous increase in net density during the analysis, thus eliminating the driving force for bulk settling.[17] **Equation S3** shows the relationship that needs to be satisfied to ensure that bulk settling does not occur, where $\rho_{net}$ is the net fluid density including the liquid and suspended particles and R is the distance from the centre of rotation.[15]

$$\frac{\delta \rho_{net}}{\delta R} \geq 0 \qquad \textbf{(S3)}$$

Once the size distribution has been obtained, the apex of the peak can be said to be the average diameter of the particle, if the distribution is narrow and symmetric, as is the case for our samples.

The relationship between the sedimentation time governed by Stokes' law and the diameter value provided by the DCS instrument can be shown through **Equation S4**, wherein t is equal to the

sedimentation time (s), $\rho_{particle}$ is the density of the particle (g $cm^{-3}$), $\rho_{fluid}$ is the density of the fluid (g $cm^{-3}$) and $d_{DCS}$ is the diameter reported by the DCS software. C is a constant that considers the solution viscosity, centrifuge speed, and cell geometry and is determined by the calibration sample, PVC.

$$t = \frac{c}{(\rho_{particle} - \rho_{fluid}) d_{DCS}^2} \quad \textbf{(S4)}$$

Due to the presence of a capping layer (either citrate or silica) the effective density of the particle is lower than that of gold, but it is not known *a priori*. Therefore, the DCS software assumes a value of 19.3 g $cm^{-3}$, i.e. the density of gold, for $\rho_{particle}$ as a reasonable first approximation. This leads to an underestimation of the particle size, i.e. $d_{DCS}$ is smaller than the actual particle size. The following section describes how this approximation is corrected for and how the thickness of the silica shell of SHINs with a gold core can be determined.

This can be done by manipulating **Equation S4**. It is essential to first determine the core diameter of the AuNPs used to make the SHINs using measurements on citrate-stabilized AuNPs from the same batch before determining the silica shell thickness. Both can be achieved by using **Equation S5** where $\rho_{fluid}$ is the fluid density (sucrose, 1.064 g $cm^{-3}$), $d_{core}$ represents the value of the gold core diameter, and s is the shell thickness, which for citrate stabilised AuNPs is 1 nm.[14]

$$t = \frac{c}{(\rho_{particle} - \rho_{fluid})(d_{core} + 2s)^2} \quad \textbf{(S5)}$$

To calculate the average effective density of the particles ($\rho_{particle}$) **Equation S6** can be used where $\rho_{core}$ is the density of the gold core (19.3 g $cm^{-3}$) and $\rho_{shell}$ is the density of the shell:[14]

$$\rho_{particle} = \frac{d_{core}^3 \rho_{core} + ((d_{core} + 2s)^3 - d_{core}^3)\rho_{shell}}{(d_{core} + 2s)^3} \quad \textbf{(S6)}$$

For citrate stabilised AuNPs, the citrate shell has a density of 1.4 g $cm^{-3}$. When using DCS to determine the shell thickness of the silica shell then $\rho_{shell}$ is equal to 2.65 g $cm^{-3}$. Due to the lower density of the silica shell compared to the density of the gold core, as the shell increases in thickness the $\rho_{particle}$ decreases which leads to an apparent decrease in the particle size.[14]

The Excel spreadsheet found in the repository uses **Equation S7** which takes into account **Equations S5** and **S6** to calculate the effective diameter that is observed at the apex of the DCS distribution curve in relation to shell thickness and core size.

$$apparent\ diameter = (d_{core} + 2s)\sqrt{\left(\frac{\left(\frac{(d_{core}^3\rho_{core} + ((d_{core}+2s)^3 - d_{core}^3)\rho_{shell})}{(d_{core}+2s)^3}\right) - \rho_{fluid}}{(\rho_{core} - \rho_{fluid})}\right)} \quad \textbf{(S7)}$$

An example of how the spread sheet is used is shown in **Figure S8**.

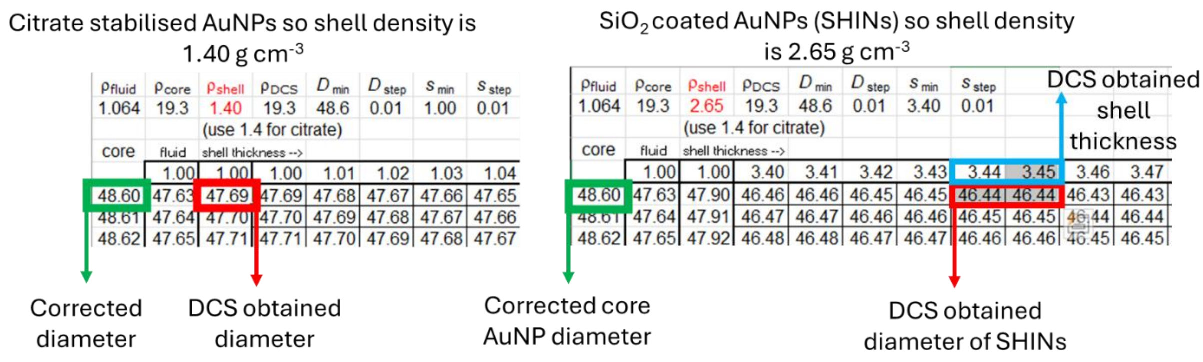


**Figure S8**. Example of using the Excel spreadsheet to ascertain the true diameter of the AuNPs and thus the shell thickness of the SHINs by using results obtained from the DCS distribution curves.

This spreadsheet calculates the diameter $d_{DCS}$ which would be reported by the DCS software for a nanoparticle with the core diameters stated in the first column and a shell with the thickness values stated in the fifth line. The shell density used in this calculation is indicated in red. (Left) Determination of the core size using DCS results for citrate-stabilised AuNPs, using a shell density of $\rho_{shell}$ = 1.4 g cm$^{-3}$. For this particular batch of AuNPs, the DCS size distribution was found to have its maximum at 47.69 nm (red), which corresponds to a core size of 48.60 nm (green) based on the reported citrate shell thickness of 1 nm.[14] (Right) Determination of the silica shell thickness from DCS results for SHINs synthesised from the same batch of AuNPs with a core diameter 48.60 nm (green), using a shell density of $\rho_{shell}$ = 2.65 g cm$^{-3}$. The maximum of the size distribution was found at 46.44 nm (red), indicating a shell thickness of 3.44-3.45 nm (blue).

### 3.2. DCS distribution curves and shell thicknesses for three different syntheses.

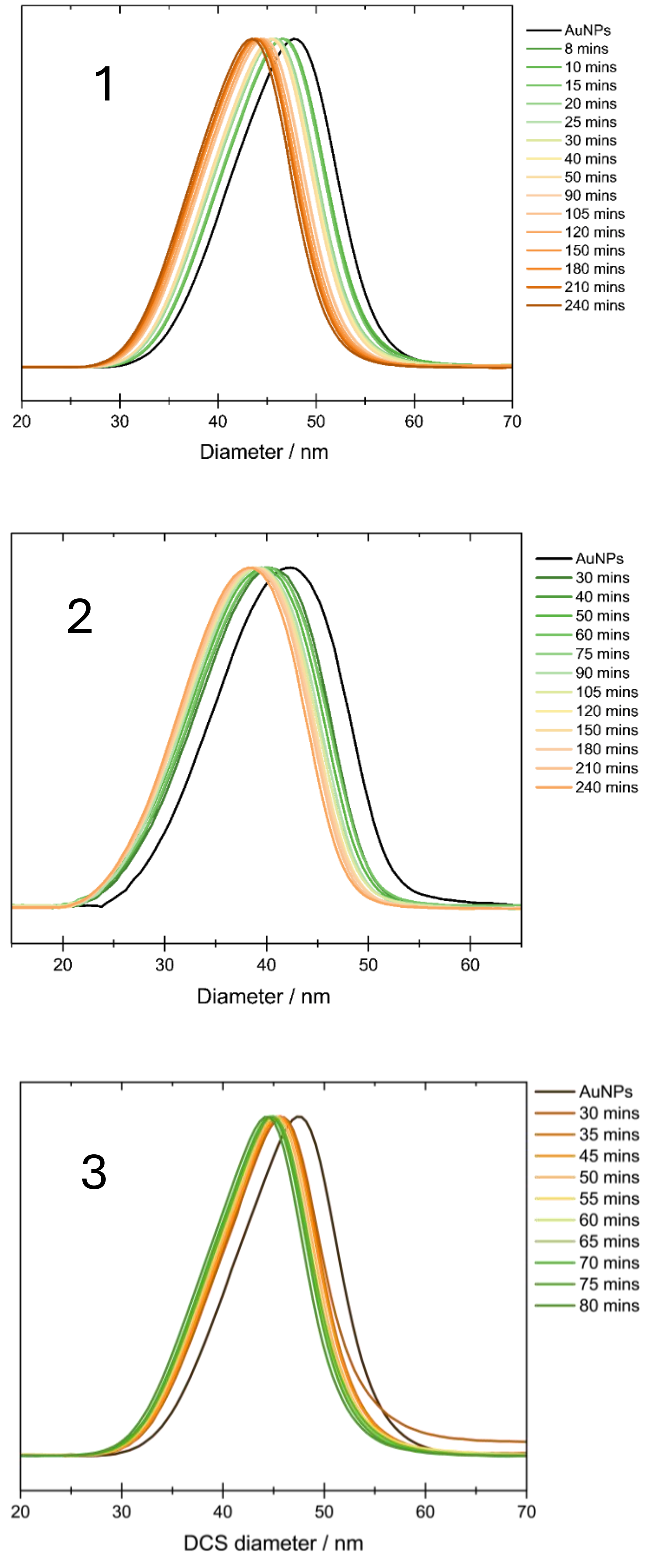


**Figure S9.** DCS results for three SHIN syntheses (1, 2, and 3) showing the DCS curves of all samples.

**Table S1.** Average shell thicknesses as determined DCS for each sample taken at separate times in three syntheses.

| 1 Synthesis time (minutes) | DCS Shell thickness (nm) | 2 Synthesis time (minutes) | Shell thickness (nm) | 3 Synthesis time (minutes) | Shell thickness (nm) |
|---|---|---|---|---|---|
| 8 | 2.88 | 30 | 4.69 | 30 | 4.26 |
| 10 | 3.07 | 40 | 6.27 | 35 | 4.53 |
| 15 | 3.25 | 50 | 7.02 | 45 | 4.66 |
| 20 | 4.32 | 60 | 5.91 | 50 | 5.36 |
| 25 | 5.03 | 75 | 8.22 | 55 | 6.38 |
| 30 | 5.23 | 90 | 8.91 | 60 | 6.62 |
| 40 | 5.14 | 105 | 8.7 | 65 | 6.89 |
| 50 | 5.93 | 120 | 10.36 | 70 | 6.24 |
| 90 | 7.31 | 150 | 10.25 | 75 | 7.2 |
| 105 | 7.61 | 180 | 10.25 | 80 | 7.78 |
| 120 | 8.72 | 210 | 12.14 | | |
| 150 | 10.04 | 240 | 15.3 | | |
| 180 | 10.39 | | | | |
| 210 | 10.76 | | | | |
| 240 | 12.77 | | | | |

### 4.1. Shell thickness determination through transmission electron microscopy (TEM)

To assess whether DCS can be used as a quick, accessible, and accurate method for determining the shell thickness of SHINs, a comparison of the DCS results to TEM images of the synthesised particles was performed. TEM uses a high-powered electron beam that is shone on a sample in a high vacuum. The electrons are transmitted through the sample and detected to form a greyscale image. The changes in the electron density of the sample causes different shades on the image.[18] The darker the shade the more dense the sample is. As the $SiO_2$ shell is less electron dense than the Au core, it will appear lighter in the image and is therefore distinguishable from the core, **Figure S9**.

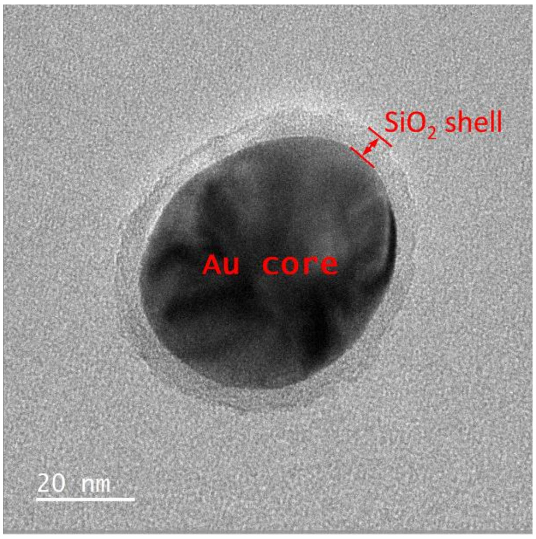


**Figure S10.** TEM image of an Au core with an $SiO_2$ shell that was synthesised for 50 minutes on a holey carbon Cu TEM grid showing the density differences between the two materials.

To determine the average shell thickness of the particles *via* TEM the obtained image requires further processing. This was done by using Digital Micrograph version 3.61. Here the line annotation tool was calibrated to that of the scale bar on the TEM image and multiple sections of the shell were measured, **Figure S10**. The lengths of these lines across the shell were then averaged. This was performed across the whole sample and then each of the average shell thicknesses of the images (*ca*. 7 – 10 images) were averaged. This was used as the average shell thickness of the whole sample as characterised by TEM.

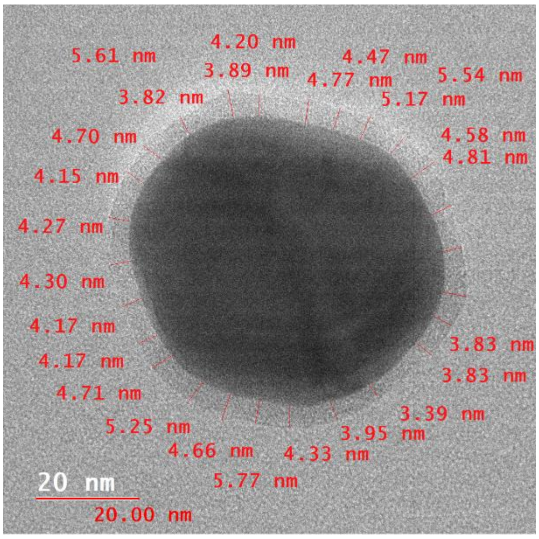


**Figure S11.** TEM image of a SHIN with an $SiO_2$ shell with annotated length lines showing the calibration of the line at the scale bar of 20 nm and the thickness of the shell at points across the shell which determined the average of the shell thickness to be 4.49 nm.

For a TEM image of a sample to be used for the analysis, the image must have at least 75% of the shell not overlapping with other SHINs and the shell must be visible. On each image used, calibrated lines were drawn from the edge of the AuNP to the edge of the shell, **Figure S10**, and this was repeated for at least 7 images. The averages of these lines were taken for each image and then averaged across all images for the sample along with the standard deviation for the sample. **Figure S11** shows just 6 of the images of a sample of SHINs taken at 30 minutes into the shell growth. This demonstrates that as much of the shell as possible was processed to create the average shell thickness for the sample. Where the shell overlaps with or is indistinguishable from the SHINs around it the thickness was not measured as this may lead to false measurements.

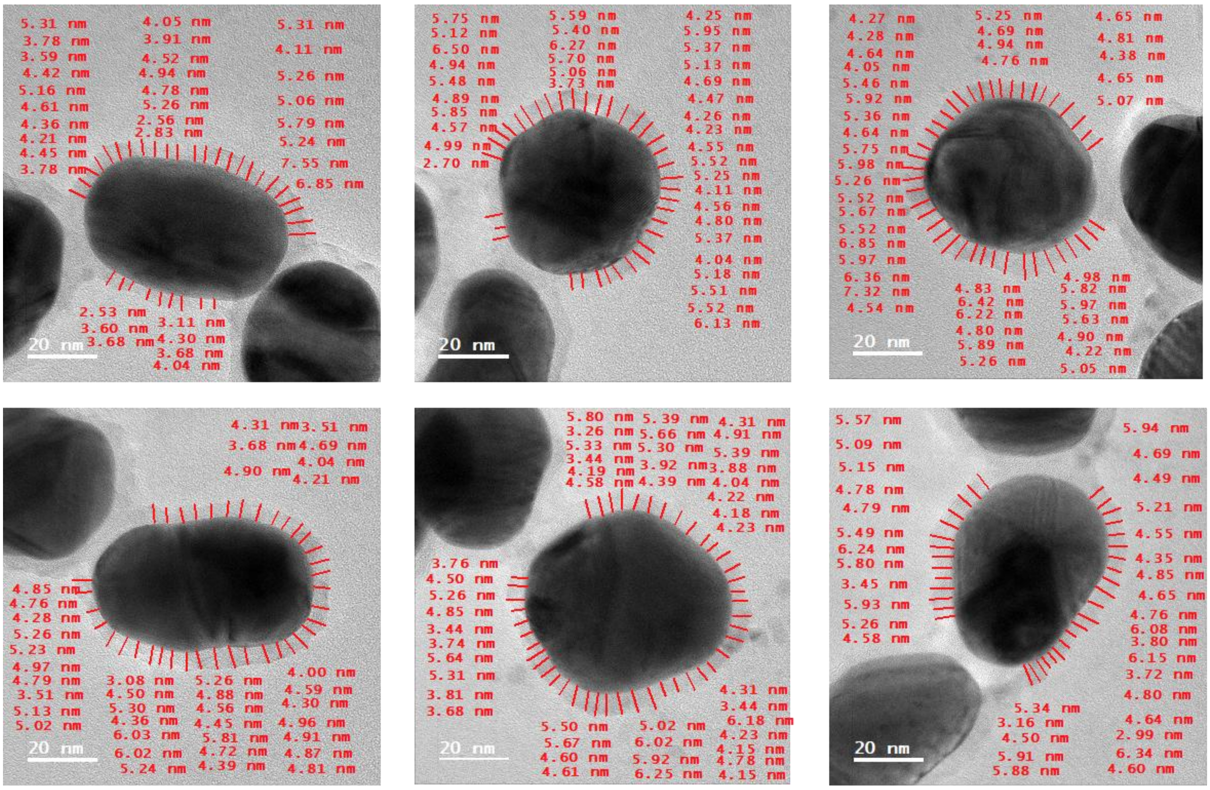


**Figure S12**. TEM images of SHINs taken after 30 minutes of shell growth with the length lines annotated to depict how each SHIN was processed. The images were taken of different SHINs from the same synthesis sample.

### 4.2. TEM images and shell thicknesses from samples of different syntheses

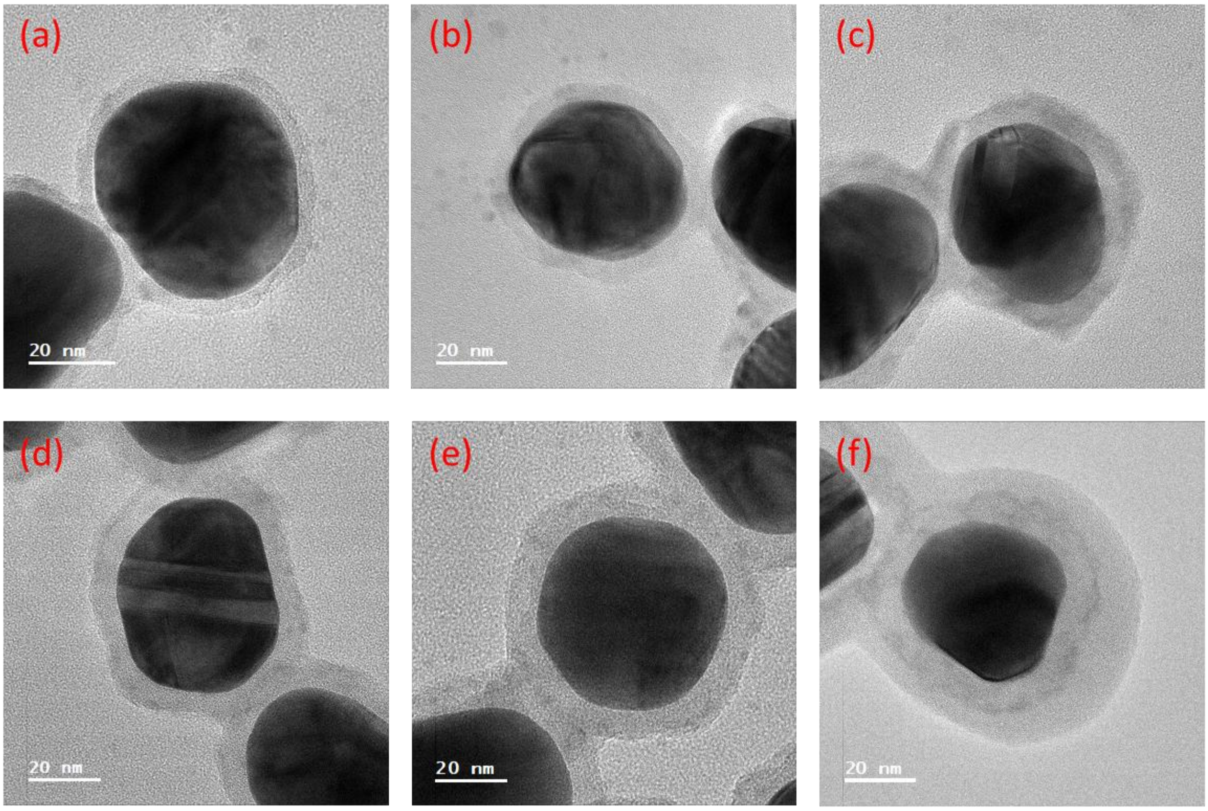


**Figure S13**. TEM images of SHINs taken at different intervals during one synthesis showing the increasing shell thickness: (a) 10 minutes, (b) 30 minutes, (c) 60 minutes, (d) 120 minutes, (e) 180 minutes, and (f) 240 minutes. There appears to be a denser silica layer in the SHIN where shell synthesis is completed for 240 minutes (f) which is shown through a darker shade line observed in the lighter shade silica coating the AuNP.[19]

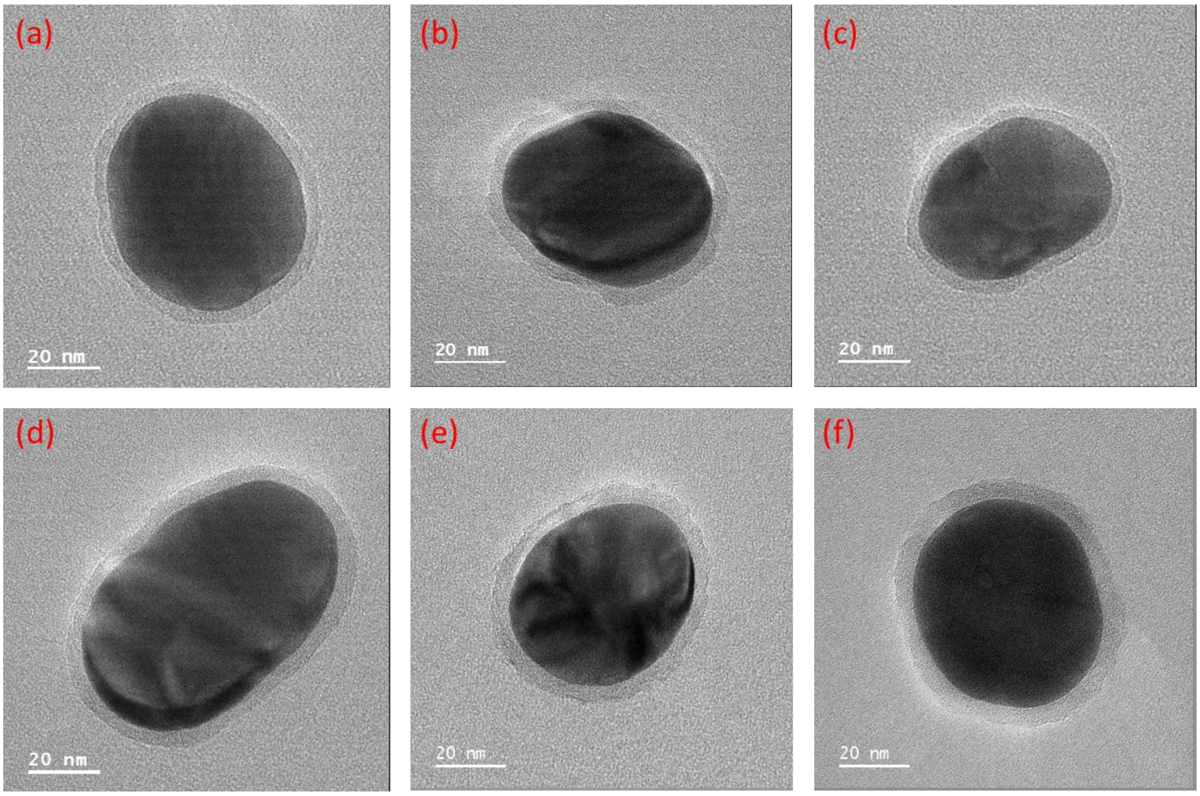


**Figure S14**. TEM images of SHINs taken at different intervals during the synthesis showing the increasing shell thickness with 5 minute intervals: (a) 30 minutes, (b) 35 minutes, (c) 45 minutes, (d) 50 minutes, (e) 55 minutes, and (f) 60 minutes. Note the 45 minute sample had aggregated before TEM analysis could be completed.

**Table S2**. average shell thicknesses as determined through the processing of multiple TEM images and the standard deviations for each sample taken at separate times across three different syntheses.

| 1 **Synthesis time (minutes)** | **TEM Shell thickness (nm) (s.t.d)** | 2 **Synthesis time (minutes)** | **Shell thickness (nm) (s.t.d)** | 3 **Synthesis time (minutes)** | **Shell thickness (nm) (s.t.d)** |
|---|---|---|---|---|---|
| 2 | 0.00 | 10 | 3.42 (0.22) | 30 | 3.89 (0.29) |
| 4 | 2.31 (0.14) | 15 | 3.82 (0.23) | 35 | 3.83 (0.19) |
| 6 | 2.69 (0.22) | 20 | 4.62 (0.35) | 45 | 4.77 (0.91) |
| 8 | 2.05 (0.37) | 25 | 4.79 (0.38) | 50 | 4.54 (0.27) |
| 10 | 2.11 (0.53) | 30 | 4.71 (0.36) | 55 | 4.76 (0.31) |
| 15 | 2.78 (0.60) | 40 | 5.04 (0.31) | 60 | 5.15 (0.28) |
| 20 | 3.23 (0.48) | 50 | 6.47 (0.51) | 65 | 5.84 (0.13) |
| 25 | 3.22 (0.58) | 60 | 7.32 (0.44) | | |
| 30 | 4.01 (0.52) | 75 | 7.32 (0.63) | | |
| 40 | 5.01 (0.26) | 90 | 8.93 (1.02) | | |
| 50 | 4.42 (0.45) | 105 | 10.68 (1.01) | | |
| 90 | 5.00 (0.63) | 120 | 9.33 (1.11) | | |
| 105 | 5.48 (0.63) | 150 | 8.12 (0.91) | | |
| 120 | 5.67 (0.22) | 180 | 9.27 (0.66) | | |
| 150 | 5.49 (0.42) | 210 | 10.37 (0.78) | | |
| 180 | 7.35 (0.31) | 240 | 13.86 (2.89) | | |
| 210 | 7.41 (0.42) | | | | |
| 240 | 8.14 (0.25) | | | | |

### 4.3. TEM images of particles exhibiting potential aggregation

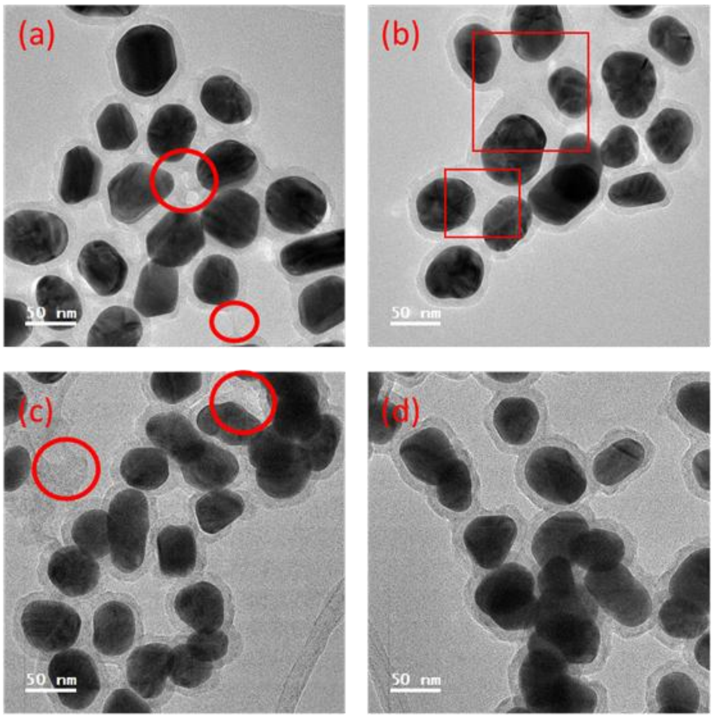


**Figure S15**. TEM images of a collection of SHINs where the samples may exhibit signs of aggregation. The red circles in (a) and (c) highlight silicate bridges and free silica within the sample. The red boxes (b) indicate areas where the nanoparticles have been fused together with silica. (d) shows a potential agglomeration of at least nine SHINs. All these observations can cause an increase in observed particle size through DCS and indicate particle interactions which can indication aggregation.

## 5. UV-Vis estimation of shell thickness

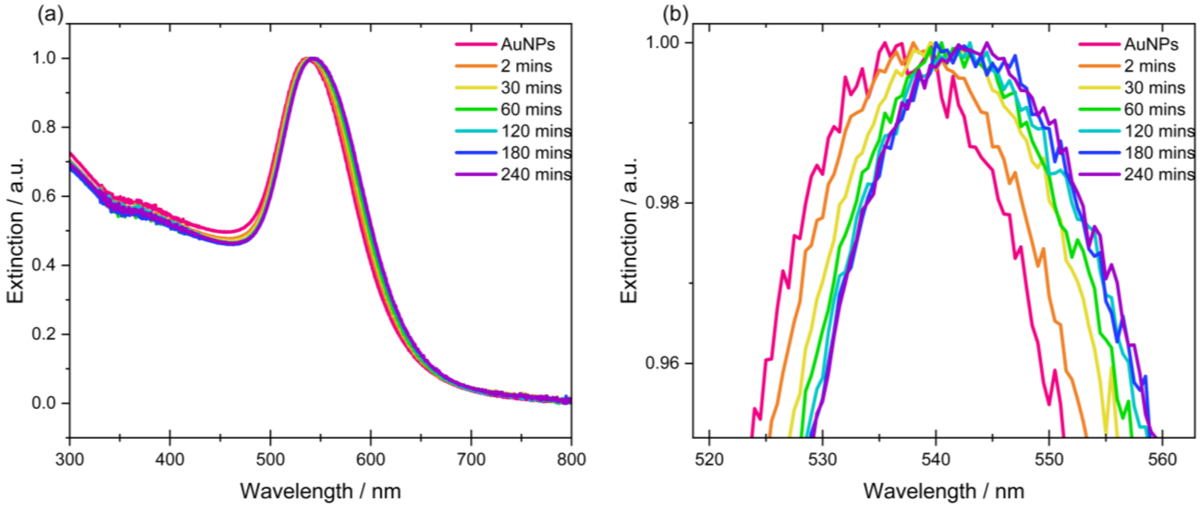


**Figure S16.** UV-Vis spectra of AuNPs before being used in a SHIN synthesis and at time intervals during a SHIN synthesis (a) and an enlarged spectrum of the maximum extinction peak showing the shift to longer wavelengths due to the shell growth (b).

By using the Haiss equation (**Equation S8**) an estimation of the citrate stabilised AuNP particle can be determined by using the LSPR band wavelength (pink trace).

$$d = \frac{\ln(\frac{\lambda_{SPR} - \lambda_0}{L_1})}{L_2} \quad \textbf{(S8)}$$

This equation can only be used with nanoparticles with a diameter ranging between 35 and 100 nm due to particles smaller than 35 nm having SPR peaks that are lower than predicted by theory due to the increase in surface atoms compared to the bulk particle, and so this equation is not valid in this case.[20] The $\lambda_{spr}$ term corresponds to the wavelength at the maximum absorbance for the nanoparticles that have been measured whereas $\lambda_0$ is equal to 512 nm. Therefore, when using this equation, the particles must be greater than 35 nm. The constants $L_1$ = 6.53 and $L_2$ = 0.0216 were all taken from Haiss *et al.*[20]

For silica-covered AuNPs (SHINs), the $\lambda_{max}$ also increases to greater wavelengths with increasing reaction time, as shown in **Figure S16**. This observed red-shift of the LSPR band with increasing shell thickness arises from the change of the dielectric constant in the immediate vicinity of the AuNP surface. Although in principle it might be possible to use this shift for determining the shell thickness, quantification of the effect requires extensive calculations based on Mie theory.

**Figure S17** and **Table S3** show that there is a correlation between increasing shell thickness and the red-shift; however, the red shift with increasing shell thickness saturates at shell thicknesses which are

smaller than the range of values investigated here. This behaviour is consistent with the distance-dependent nature of LSPR effects, where only the dielectric environment within several nanometres of the Au surface significantly influences the LSPR band position. Consequently, once the silica shell exceeds this interaction region, further shell growth produces only minor spectral shifts despite the continued increase in the physical shell thickness measured by DCS and TEM.

Therefore, we affirm that UV-vis spectrophotometry is of limited use for a precise analysis of the shell thickness of SHINs.

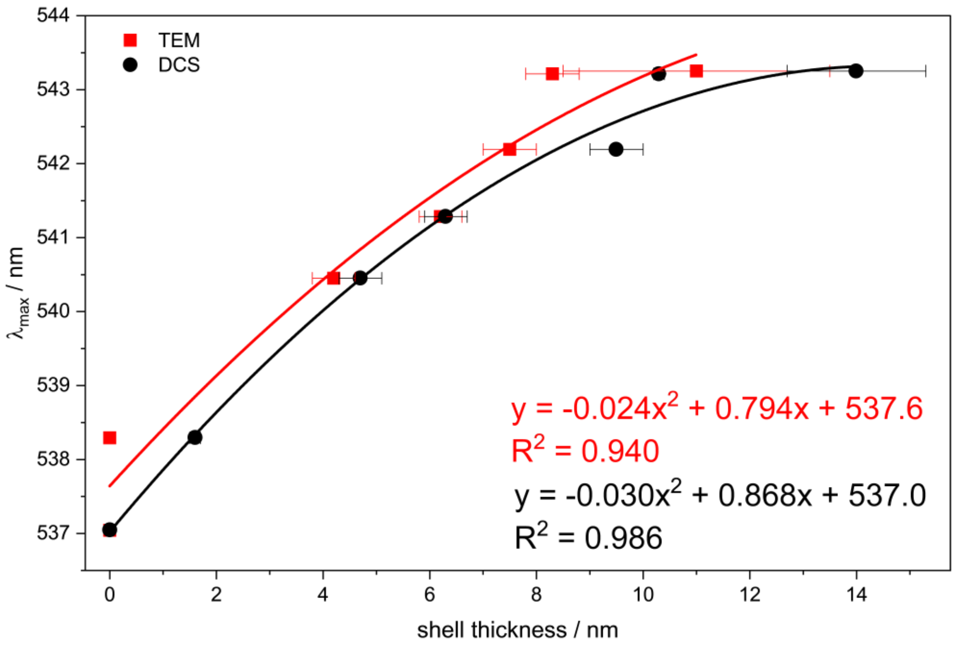


**Figure S17.** Extinction maxima ($\lambda_{max}$) as a function of average shell thickness determined by DCS (red) and TEM (black), with lines of best fit to a polynomial of second order and corresponding $R^2$ values shown in the respective colours.

**Table S3**. average shell thicknesses as determined through DCS and TEM and the standard deviations for each and the associated $\lambda_{max}$ as determined through UV-Vis in **Figure S15**.

| $\lambda_{max}$ | shell thickness TEM (nm) | shell thickness DCS (nm) |
|---|---|---|
| 537.04 | 0 | 0 |
| 538.29 | 0 | 1.6 (0.1) |
| 540.45 | 4.2 (0.4) | 4.7 (0.4) |
| 541.28 | 6.2 (0.4) | 6.3 (0.4) |
| 542.19 | 7.5 (0.8) | 9.5 (0.5) |
| 543.22 | 8.3 (0.5) | 10.3 (0.1) |
| 543.25 | 11.0 (2.5) | 14.0 (1.3) |

## 6. Confidence interval evaluation of DCS and TEM derived shell thicknesses

By calculating the confidence intervals (CI) between the DCS and TEM derived shell thicknesses the deviation between TEM and DCS can be evaluated and understood if it is statically relevant or not. This was done by using a simple confidence interval calculation using a 95% confidence and assuming the shell thicknesses are normally distributed.

The CI analysis indicates that at shorter synthesis times the shell thicknesses obtained via DCS and TEM agree within experimental uncertainty. However, for thicker shells (obtained at longer synthesis times), the CI no longer consistently overlap (see **Table S4**), suggesting that the divergence exceeds what would be expected from sampling variability alone and that systematic effects contribute to the discrepancy.

**Table S4.** average DCS and TEM shell thicknesses in nm over 3 syntheses at various times in minutes and the calculated confidence interval (CI). DCS/TEM-CI shows the value of shell thickness with the CI subtracted and DCS/TEM+CI shows the value of the shell thickness with the CI added. Highlighted rows indicate the CIs that overlap.

| Time / s | DCS (nm) | CI (±) | TEM (nm) | CI (±) |
|---|---|---|---|---|
| 120 | 9.50 | 1.09 | 7.50 | 1.09 |
| 150 | 10.20 | 0.14 | 6.80 | 0.95 |
| 180 | 10.30 | 0.14 | 8.30 | 0.68 |
| 210 | 11.50 | 0.95 | 8.90 | 0.82 |
| 240 | 14.00 | 1.77 | 11.00 | 2.85 |
| **Time / s** | **DCS-CI** | **DCS+CI** | **TEM-CI** | **TEM+CI** |
| 120 | 8.41 | 10.59 | 6.41 | 8.59 |
| 150 | 10.06 | 10.34 | 5.85 | 7.75 |
| 180 | 10.16 | 10.44 | 7.62 | 8.98 |
| 210 | 10.55 | 12.45 | 8.08 | 9.72 |
| 240 | 12.23 | 15.77 | 8.15 | 13.85 |

The observed discrepancy between TEM- and DCS-derived shell thicknesses at longer synthesis times is however consistent with the physical basis of DCS. DCS determines an apparent hydrodynamic/sedimentation diameter that depends on the effective particle density (described by Stokes-based sedimentation models presented in **Eq. S5-S7**). As the $SiO_2$ shell becomes thicker, structural heterogeneity, hydration, porosity, and/or lower shell condensation can reduce the effective density of the shell region. This decreases the sedimentation coefficient and shifts the apparent DCS diameter, resulting in a larger calculated shell thickness when a constant $SiO_2$ density is assumed.

This interpretation is consistent with previous reports that describe density distributions and effective density corrections in core-shell nanoparticle sedimentation analyses.[21-23] Furthermore, TEM provides

local measurements of a relatively small subset of particles, whereas DCS probes the ensemble-average behaviour of the nanoparticle population in suspension. The increasing discrepancy at larger shell thicknesses therefore likely reflects both changes in effective particle density and differences between local and ensemble-sampling methodologies.[21, 23]

**References**


1. D. Raciti, T. Braun, A. R. Hight Walker and T. P. Moffat, *Journal of The Electrochemical Society*, 2022, **169**, 082506.
2. X.-D. Tian, B.-J. Liu, J.-F. Li, Z.-L. Yang, B. Ren and Z.-Q. Tian, *Journal of Raman Spectroscopy*, 2013, **44**, 994–998.
3. J. F. Li, Y. F. Huang, Y. Ding, Z. L. Yang, S. B. Li, X. S. Zhou, F. R. Fan, W. Zhang, Z. Y. Zhou, D. Y. Wu, B. Ren, Z. L. Wang and Z. Q. Tian, *Nature*, 2010, **464**, 392–395.
4. L. M. Liz-Marzán, M. Giersig and P. Mulvaney, *Langmuir*, 1996, **12**, 4329–4335.
5. J. Turkevich, *Gold Bulletin*, 1985, **18**, 86–91.
6. G. Frens, *Nature Physical Science*, 1973, **241**, 20–22.
7. S. Bharathi and O. Lev, *Chemical Communications*, 1997, DOI: 10.1039/a705609e, 2303–2304.
8. R. D. Deegan, O. Bakajin, T. F. Dupont, G. Huber, S. R. Nagel and T. A. Witten, *Nature*, 1997, **389**, 827–829.
9. A. Kaliyaraj Selva Kumar, Y. Zhang, D. Li and R. G. Compton, *Electrochemistry Communications*, 2020, **121**, 106867.
10. T. A. Galloway and L. J. Hardwick, *The Journal of Physical Chemistry Letters*, 2016, **7**, 2119–2124.
11. T. A. Galloway, L. Cabo-Fernandez, I. M. Aldous, F. Braga and L. J. Hardwick, *Faraday Discussions*, 2017, **205**, 469–490.
12. J. Fernández-Vidal, A. M. Gómez-Marín, L. A. H. Jones, C.-H. Yen, T. D. Veal, V. R. Dhanak, C.-C. Hu and L. J. Hardwick, *The Journal of Physical Chemistry C*, 2022, **126**, 12074–12081.
13. S. Moran, in *An Applied Guide to Water and Effluent Treatment Plant Design*, ed. S. Moran, Butterworth-Heinemann, 2018, DOI: https://doi.org/10.1016/B978-0-12-811309-7.00005-9, pp. 53–58.
14. Ž. Krpetić, A. M. Davidson, M. Volk, R. Lévy, M. Brust and D. L. Cooper, *ACS Nano*, 2013, **7**, 8881–8890.
15. D. J. Scott, S. E. Harding, A. J. Rowe and R. S. o. Chemistry, *Analytical Ultracentrifugation: Techniques and Methods*, RSC Publishing, 2005.
16. C. F. Bohren and D. R. Huffman, *Absorption and Scattering of Light by Small Particles*, Wiley, 2008.
17. K. L. Kelly, E. Coronado, L. L. Zhao and G. C. Schatz, *The Journal of Physical Chemistry B*, 2003, **107**, 668–677.
18. B. Misof, P. Roschger and P. Fratzl, in *Comprehensive Biomaterials*, ed. P. Ducheyne, Elsevier, Oxford, 2011, DOI: https://doi.org/10.1016/B978-0-08-055294-1.00112-4, pp. 407–426.
19. W. Yang, S. Kaur, Y. D. Kim, J.-M. Kim, S. H. Lee and D.-K. Lim, *Journal of Materials Chemistry B*, 2022, **10**, 364–372.
20. W. Haiss, N. T. K. Thanh, J. Aveyard and D. G. Fernig, *Analytical Chemistry*, 2007, **79**, 4215–4221.
21. A. M. Davidson, M. Brust, D. L. Cooper and M. Volk, *Analytical Chemistry*, 2017, **89**, 6807–6814.
22. C. S. Plüisch, R. Stuckert and A. Wittemann, *Journal*, 2021, **11**, 1027.

23. L. A. Fielding, O. O. Mykhaylyk, S. P. Armes, P. W. Fowler, V. Mittal and S. Fitzpatrick, *Langmuir*, 2012, **28**, 2536–2544.
